# Macrophage-derived CCL20 promotes abdominal aortic aneurysm progression via lymphocytes CCR6

**DOI:** 10.3389/fimmu.2026.1780720

**Published:** 2026-03-02

**Authors:** Qingnan Ren, Tianyong Sun, Song Shen, Yuanbin Cao, Li Wei, Yang Zhao, Fengxin Wan, Ping Sui, Ke Xiao, Hao Bai, Dachuan Guo, Qi He, Mengfan Zhi, Jianmin Yang, Jianjun Jiang, Wencheng Zhang, Xiangjiu Ding

**Affiliations:** 1 Department of Vascular Surgery, General Surgery, Qilu Hospital of Shandong University, Jinan, Shandong, China; 2 Department of Health Care (Department of General Dentistry II), Human Microbiome, Shandong Key Laboratory of Oral Tissue Regeneration, Shandong Engineering Research Center of Dental Materials and Oral Tissue Regeneration, Shandong Provincial Clinical Research Center for Oral Diseases, School and Hospital of Stomatology, Cheeloo College of Medicine, Shandong University, Jinan, Shandong, China; 3 Department of Clinical Laboratory, Qilu Hospital of Shandong University, Jinan, Shandong, China; 4 Clinical Epidemiology Unit, Department of Nutrition, Clinical Research Center of Shandong University, Qilu Hospital of Shandong University, Jinan, Shandong, China; 5 Department of Cardiology, State Key Laboratory for Innovation and Transformation of Luobing Theory, Key Laboratory of Cardiovascular Remodeling and Function Research of MOE, NHC, CAMS and Shandong Province, Qilu Hospital of Shandong University, Jinan, Shandong, China; 6 Shandong Key Laboratory of Medicine and Prevention Integration in Rheumatism and Immunity Disease, Qilu Hospital of Shandong University, Jinan, Shandong, China

**Keywords:** abdominal aortic aneurysm, C-C motif chemokine ligand 20, C-C motif chemokine receptor 6, CCL20-CCR6 axis, vascular disease

## Abstract

**Introduction:**

Abdominal aortic aneurysm (AAA) is a chronic vascular disease marked by chronic inflammation and immune dysregulation. The C-C motif chemokine ligand 20 (CCL20) - C-C motif chemokine receptor type 6 (CCR6) axis modulates immune responses in vascular diseases, but its role in AAA remains unclear. This study investigates the involvement of the CCL20-CCR6 axis in AAA formation.

**Methods:**

Single-cell RNA sequencing datasets and bulk RNA sequencing datasets were analyzed to assess cellular composition and transcriptional changes. Transcriptomic analysis, enzyme-linked immunosorbent assay, UK Biobank database analysis, CellChat analysis, immunofluorescence staining, and mouse models were employed to explore the CCL20-CCR6 axis in AAA.

**Results:**

Substantial immune cell infiltration (T lymphocytes & B lymphocytes) and loss of structural cells (fibroblasts, endothelial cells and smooth muscle cells) were identified using single-cell RNA sequencing datasets. Macrophage polarization was imbalanced, with enriched M1-like macrophages and elevated CCL20 secretion. Macrophages could promote the formation of AAA by recruiting a large number of immune cells via the CCL20-CCR6 axis. *In vitro*, CCL20 neutralization reduced immune cell recruitment; *in vivo*, the knockdown of this axis inhibited AAA progression.

**Conclusions:**

Macrophage-derived CCL20 aggravates lymphocyte recruitment via the CCR6, promoting AAA progression. CCL20 may serve as a biomarker for AAA. Targeting the CCL20-CCR6 axis could inhibit immune recruitment and AAA progression.

## 1 Introduction

Abdominal aortic aneurysm (AAA) represents a progressive, life-threatening vascular condition characterized by irreversible dilation of the abdominal aorta, often culminating in rupture. Epidemiological data indicate an incidence of 2-8% among individuals over 65 years, with rupture-associated mortality reaching 24.5% despite advancements in endovascular repair (1–3). Current clinical strategies rely predominantly on surgical interventions, as effective pharmacological therapies remain elusive, underscoring the urgent need for novel therapeutic targets to mitigate AAA progression and improve patient outcomes.

The pathogenesis of AAA is intricately linked to chronic inflammation, immune dysregulation, and structural degradation of the aortic wall. Key hallmarks include macrophage polarization, apoptosis of vascular smooth muscle cells (SMCs), and extracellular matrix remodeling (4–6). Emerging evidence highlights the pivotal role of immune cells in driving this inflammatory cascade, with macrophages acting as key regulators (7–9). Specifically, a variety of immune cells contribute to AAA progression in both human tissues and Angiotensin II (Ang II)-induced *Apolipoprotein E-/- (ApoE-/-)* mice models (10–12), including macrophages (13–15), T lymphocytes (T cells) (13, 14), and B lymphocytes (B cells) (16, 17). Macrophage-mediated immune regulation is pivotal in AAA progression (8, 18), where M1 macrophage polarization and associated inflammation drive aneurysm formation via proteolytic enzymes and pro-inflammatory cytokines (18–20). This polarization imbalance, triggered by environmental cues, sustains chronic inflammation and disrupts M1/M2 equilibrium (21, 22).

Within AAA tissues, macrophages interact closely with T and B cells (17, 23), facilitating immune cell communication and recruitment. Macrophages enhance T-cell infiltration through cytokine and chemokine signaling (24), while T cells, as predominant infiltrates in AAA (25), amplify progression via cytokine secretion (26–28). B cells contribute to AAA progression through immunoglobulin production and cytokines (16, 29), and their depletion in mice attenuates aneurysms by fostering immunosuppression (30, 31). However, the full mechanisms of immune cell infiltration and interactions in AAA remain unclear (32), necessitating further investigation for deeper insights.

C-C chemokine ligand 20 (CCL20), also known as macrophage inflammatory protein-3α, is produced by multiple cell types in the vascular wall, including activated endothelial cells, SMCs, stromal cells, and infiltrating monocytes/macrophages, particularly under inflammatory conditions (33–35). Through its interaction with its exclusive receptor, C-C motif chemokine receptor type 6 (CCR6), which is expressed on T cells, B cells, Natural Killer T (NKT) cells, and neutrophils (33, 36, 37), CCL20 mediates the recruitment of these immune cells to inflammatory sites (38, 39). The CCL20-CCR6 axis has garnered significant interest in recent years, as evidenced by studies establishing its functional involvement in vascular pathologies (37, 40, 41). However, the relationship between the CCL20-CCR6 axis and the pathogenesis of AAA has yet to be fully understood.

This study integrates single-cell RNA sequencing datasets and bulk RNA sequencing datasets with experimental validations to reveal how macrophage polarization imbalances promote immune recruitment and inflammation via the CCL20-CCR6 axis. Overall, these findings offer novel perspectives on immune regulation in AAA, positioning the CCL20-CCR6 axis as a critical contributor to AAA progression. The intervention experiments are employed to investigate the role and mechanism of this axis in AAA progression, with the aim to provide valuable insights into the immune regulatory mechanisms and therapeutic potential of the CCL20-CCR6 axis in AAA pathogenesis and progression.

## 2 Materials and methods

### 2.1 Collection of human abdominal aortic tissue and peripheral blood samples

This study was conducted in accordance with the principles of the Declaration of Helsinki and received approval from the Ethics Committee of Qilu Hospital of Shandong University (Approval No.: KYLL-202503-09-046-1) for human samples. Written informed consent was obtained from all participants or the families of organ donors. Human abdominal aortic aneurysm (AAA) tissues were collected from six patients (4 males, 2 females; age range: 58–71 years, mean: 65.83 years, median: 67.5 years, standard deviation(SD) = 5.27, interquartile range (IQR): 10) undergoing open surgical repair for AAA. Normal aortic tissues were obtained from five organ donors (5 males; age range: 51–74 years, mean: 58 years, median: 56 years, standard deviation(SD) = 9.92, interquartile range (IQR): 17) who had died from cerebral hemorrhage. The demographics and comorbidities of the AAA patients and organ donors are summarized in **Supplementary Table 1**. These tissues were used for Western blot, immunohistochemistry (IHC), immunofluorescence (IF) staining and multiplex immunofluorescence (mIF).

Serum samples were collected from 80 patients with AAA and 79 healthy controls. Inclusion criteria for AAA patients included an AAA diameter exceeding 30 mm and a definite diagnosis of AAA confirmed by computed tomography angiography of the full aorta. Exclusion criteria for AAA patients encompassed false (infected, inflammatory, or traumatic) or dissected AAAs, those caused by genetic or connective tissue diseases such as Ehlers-Danlos syndrome or Marfan syndrome, a previous history of open or endovascular repair for AAAs, malignant tumors, acute or chronic infections, autoimmune diseases, and current use of antibiotics, anti-inflammatory drugs, or immunosuppressive therapy. The control group consisted of healthy individuals matched for age and gender. Serum samples from AAA patients were obtained from the Department of Vascular Surgery at Qilu Hospital of Shandong University, while those from controls were collected from the Department of Health Management Center at the same institution. Demographic and clinical information for both groups is summarized in **Supplementary Table 2**. All samples were stored at 4°C until analysis.

### 2.2 Animal experiments

All animal procedures followed the National Institutes of Health *Guide for the Care and Use of Laboratory Animals* and were approved by the Experimental Animal Ethics Committee of Qilu Hospital of Shandong University (Approval No.: DWLL-2024-214). Eight-week-old *ApoE^-/-^* male mice (C57BL/6J background) were obtained from Vital River Laboratory (China) and housed in pathogen-free conditions (12-h light/dark cycle, 24 ± 2°C, 40 ± 5% humidity, five mice per cage). The mice were fed with a Western diet for four weeks to induce the formation of AAA. The western-style diet (high cholesterol diet) consisted of 17.3% protein, 21.2% fat, 48.5% carbohydrates, 0.2% cholesterol by mass, contributing 42% of total calories from fat (TD.88137, Envigo).

### 2.3 Angiotensin II-induced mice AAA model

To induce AAA, osmotic pumps (RWD Life Science, model 2004w, China) delivering Ang II (1000 ng/kg/min, Sigma-Aldrich, USA) were subcutaneously implanted in 8-week-old *ApoE^-/-^* male mice (42). A total of 75 mice were randomly assigned to 5 experimental groups (n=15 per group): 1) Negative Control group; 2) AAV-shRNA (empty vector) group; 3) AAA model group; 4) AAV-shCCL20-treated AAA model group; and 5) AAV-CCR6-treated AAA model group. Mice were monitored daily for activity and mortality; aortic diameter was assessed weekly via Doppler ultrasound (FUJIFILM Visual Sonics, Japan). After 4 weeks, mice were euthanized with Carbon Dioxide (CO_2_), and tissues were harvested.

### 2.4 AAV-mediated CCL20/CCR6 intervention *In Vivo*

To investigate the role of CCL20/CCR6 in AAA development, *ApoE^-/-^* mice received tail vein injections of Adeno-Associated Viruses (AAV) carrying AAV9-cmv-shCCL20, AAV9-cmv-shCCR6, or a negative control (2×10^-11^vector genomes). ShRNA sequences and serotype AAV9 viruses carrying these sequences or a negative control were purchased from Cyagen (China). The target shCCL20 sequence was: 5’-CCAAAGCAGAACTGGGTGAAA-3’; the target shCCR6 sequence was: 5’-GATCCATGACTGACGTCTACCT-3’.

### 2.5 Blood pressure measurement

Mouse systolic blood pressure, diastolic blood pressure, and heart rate were measured using a non-invasive tail-cuff system (BP-98A, Softron, Japan) pre- and post-model induction and during the modeling period.

### 2.6 Ultrasound examination

AAA was defined as a ≥ 50% dilation of the abdominal aortic diameter compared to the normal diameter in mice. Vascular Doppler ultrasound provided a rapid, effective, and non-invasive method for monitoring abdominal aortic diameter. Mice were anesthetized with 2% isoflurane, and hair was removed from the chest to abdomen using depilatory cream. Ultrasound coupling gel was applied to the abdomen of each mouse. Using an Doppler ultrasound system (Visual Sonics Vevo 2100, FUJIFILM, Japan) equipped with a 40 MHz transducer, the probe position was adjusted to identify the abdominal aorta via B-mode ultrasound. Color and spectral Doppler were used to confirm the blood direction and velocity consistent with aortic signals. The abdominal aortic diameter was measured three times per mouse via B-mode, color, and spectral Doppler.

### 2.7 Histopathological analysis

Mouse aortas were fixed in 4% paraformaldehyde for 24 hours, dehydrated through a graded ethanol series, cleared in xylene, embedded in paraffin, and sectioned into 5 μm serial slices. After standard deparaffinization and rehydration, the sections were processed as follows: Oil Red O staining for lipid deposition, hematoxylin and eosin staining (Sigma-Aldrich, USA) for histopathological changes and vascular integrity, elastic Van Gieson staining (Sigma-Aldrich, USA) for elastic fiber fragmentation, and Masson’s trichrome staining (Solarbio, China) for collagen deposition. Stained sections were observed under a microscope (Nikon Eclipse E100, Japan) and analyzed quantitatively using ImageJ software (three fields per sample, double-blinded). Elastic degradation was graded as follows: Grade 1, intact with no degradation; Grade 2, mild elastic degradation; Grade 3, severe elastic degradation; Grade 4, aortic rupture.

### 2.8 Immunohistochemistry

IHC was performed using an IHC kit (Solarbio, China). Sections were deparaffinized, subjected to heat-induced ethylenediaminetetraacetic acid (EDTA) epitope retrieval, blocked with goat serum, and incubated overnight at 4°C with primary antibodies: CD3 (Abcam, ab16669, 1:200, UK), CD19 (Abcam, ab245235, 1:200, UK), and CD68 (Abcam, ab282654, 1:150, UK). After washing with phosphate-buffered saline three times (5 minutes each), sections were incubated with biotinylated secondary antibodies, stained with 3,3’ -diaminobenzidine, and counterstained with hematoxylin. Stained sections were imaged under a microscope (Nikon Eclipse E100, Japan), and CD3+/CD19+/CD68+ cells were quantified by two independent pathologists blinded to the groups. Ten random high-power fields (50 × magnification) per section were selected for positive cell counting.

### 2.9 Immunofluorescence staining and multiplex immunofluorescence

Human and mouse aortic tissues were fixed in 10% neutral buffered formalin, paraffin-embedded, and sectioned. Sections were deparaffinized in xylene and graded ethanol, subjected to heat-induced EDTA epitope retrieval, and blocked with goat serum for 1 hour. Sections. They were incubated overnight at 4°C with primary antibodies: CD3 (Abcam, ab16669, 1:200, UK), CD19 (Abcam, ab245235, 1:200, UK), CCL20 (Proteintech, 26527-1-AP, 1:200, China), CD68 (Abcam, ab282654, 1:150, UK), CCR6 (Abcam, ab303672, 1:150, UK), CD204 (Abcam, ab314227, 1:150, UK), and CD86 (Cell Signaling Technology, 91882T, 1:100, USA). After washing, sections were incubated with secondary antibodies at room temperature for 1 hour: Alexa Fluor 488 anti-rabbit (Proteintech, RGAR002, 1:200, China), Alexa Fluor 594 anti-rabbit (Proteintech, RGAR002, 1:200, China), and Alexa Fluor 555 anti-rabbit (Proteintech, RGAR003, 1:200, China).

Multiplex immunofluorescence was performed on formalin-fixed, paraffin-embedded (FFPE) tissue sections using the Tyramide Signal Amplification (TSA) technique. Each round consisted of the following cycle: incubation with a primary antibody, followed by an HRP-conjugated secondary antibody, and then the corresponding TSA fluorescent dye (Opal series): CD68 (Genetech, GM087629, 1:200, China, PPD 520), CD86 (Cell Signaling Technology, 19589S, 1:100, USA, PPD 570), CD206 (Cell Signaling Technology, 24595, 1:100, USA, PPD 570), CD163 (Abcam, Ab182422, 1:150, UK, PPD 620), iNOS (Bioss Antibodies, BS-0162R,1:200, China, PPD 620), Arg1 (Abclonal biotechnology, A25808, 1:200, USA, PPD 620), F4/80 (Cell Signaling Technology, 70076S, 1:100, USA, PPD 520). Nuclei were counterstained with DAPI, and stained sections were examined under a confocal fluorescence microscope (Zeiss LSM 780, Germany).

### 2.10 Enzyme-linked immunosorbent assay

Serum CCL20 levels in AAA patients and healthy controls were quantified using a commercial ELISA kit (Proteintech, KE00149, China), following the manufacturer’s protocol. Absorbance was measured at 450 nm.

### 2.11 Real-time quantitative PCR

Total RNA was extracted from tissues using a Tissue DNA Extraction Kit (TIANGEN, China). cDNA was synthesized, mixed with SYBR^®^ Green (Accurate Biology, AG11701, China) and primers, and amplified per the manufacturer’s instructions. Relative gene expression was calculated using the 2^^−ΔΔCt^ method with *GAPDH* as the reference gene. Primer sequences are shown in **Supplementary Table 3**.

### 2.12 Western blot analysis

Aortic tissue proteins were extracted using radioimmunoprecipitation assay lysis buffer, separated by sodium dodecyl sulfate-polyacrylamide gel electrophoresis, and transferred to polyvinylidene fluoride membranes. Membranes were blocked with 5% skim milk, incubated overnight at 4°C with primary antibodies (CCR6, abcam, ab227036 & GAPDH, Proteintech, 60004-1-Ig), washed with Tris-Buffered Saline with Tween 20 buffer, incubated with secondary antibodies, and detected using an enhanced chemiluminescence reagent on a chemiluminescence imaging system (Azure Biosystems, USA). Quantification was performed using the ImageJ software for digital image analysis.

### 2.13 Transwell assay

A Transwell assay was performed to evaluate the chemotactic ability of macrophages toward B and T cells under inflammatory conditions. Mouse bone marrow-derived macrophages were stimulated with lipopolysaccharide (LPS) (100 ng/mL, L2880, Sigma-Aldrich, USA) for 24 hours, and the cell supernatant was collected. Using Transwell chambers (0.4 μm pore size, LabSelect, 14112), 2×10^5^ B or T cells were seeded in the upper chamber with serum-free medium, and supernatant was added to the lower chamber. After 48 hours, migrated cells were analyzed by flow cytometry.

### 2.14 Single-cell data analysis

Single-cell data used in this study were searched and downloaded from the Gene Expression Omnibus (GEO) database (https://www.ncbi.nlm.nih.gov/geo/) under the accession numbers GSE166676 and GSE226492. The UMI expression matrix (10 × files) with genes expressed in at least 5 cells and cells expressing at least 200 genes was loaded into Seurat (version 5.3.0) (43). Further cleaning steps were performed using nCount within twofold standard deviation, nFeature within twofold standard deviation, and mitochondria percentage below 15%. Mitochondrial genes, ribosomal genes, and the MALAT1 gene were then removed from the expression dataset to minimize technical noise during downstream normalization and dimensionality reduction. The SCTransform function was used for data normalization, scaling, and transformation, with S.Score and G2M.Score (calculated using the CellCycleScoring function based on S- and G2/M-phase-specific genes) included as covariates to regress out cell cycle-associated variation. DoubletFinder (version 2.0.6) was then used for the identification and removal of double cells (44). The R package scCDC (version 1.4) was used for the gene-specific contamination detection and correction in single-cell RNA-seq data (45).

After data merging, dimensionality reduction was performed using the RunPCA function, and the number of retained principal components was determined using a quantitative elbow-based approach. PCs were retained when individual variance explained fell below 5% while cumulative variance exceeded 90%, and when the change in variance explained between consecutive PCs was <0.1%. The final PC number (14) was conservatively selected as the minimum value satisfying these criteria. To correct for batch effects between the two independent GEO datasets, Harmony integration (R package harmony, version 1.2.4) was then applied to the PCA embeddings using dataset identity as the batch variable. The Harmony-corrected low-dimensional representations were subsequently used for FindNeighbors and UMAP analyses.

The cell clusters were identified using the FindClusters function with a resolution of 0.7 and the Leiden algorithm. Marker genes for each cluster were identified using the FindAllMarkers function with the Wilcoxon rank-sum test under the following criteria: avg_log2FC > 0.25, p_val_adj < 0.05, pct1 > 0.1, and pct2 < 0.5. This analysis was performed for cluster marker identification and cell-type annotation purposes, rather than for donor-level differential expression inference. The AddModuleScore function was used to calculate module scores for predefined feature expression programs (e.g., M1-like or M2-like macrophage signatures) at the single-cell level.

### 2.15 Ro/e analysis

To assess the distributional preference of each cell cluster across different tissues or samples, we calculated the observed-to-expected ratio (Ro/e = Observed/Expected) using the R package Startrac (version 0.1.0) (46). In this analysis, cells were pooled within each condition, and the expected distribution was defined under the assumption of independence between cell cluster identity and tissue category, estimated from the marginal distributions of all pooled cells. Statistical significance was evaluated using Fisher’s exact tests as implemented in Startrac, with Benjamini–Hochberg correction applied for multiple testing. Cell clusters with adjusted P values < 0.05 were considered to show significant deviation from the expected distribution.

A Ro/e value greater than 1 indicates that a given cell cluster is overrepresented in a particular tissue relative to the expected distribution, whereas a Ro/e value less than 1 denotes relative underrepresentation. We note that this Ro/e analysis was performed at the cell level and does not explicitly account for donor-level structure. Due to the limited number of donors and sparse representation of certain cell clusters across individual samples, donor-aware Ro/e analysis was not statistically feasible. Therefore, Ro/e results are presented as a descriptive summary of cell-type distribution patterns rather than as formal donor-level inference and should be interpreted with appropriate caution.

### 2.16 Cell-cell communication analysis using CellChat

Cell-cell communication analysis was performed using the R package CellChat (version 2.2.0) (47), which infers putative intercellular communication networks based on the expression of known ligand-receptor pairs. Normalized single-cell expression data and annotated cell types were used to estimate communication probabilities between cell populations. CellChat analysis was conducted on pooled single-cell datasets within each condition to characterize global communication patterns. Inferred interaction strength and pathway-level signaling patterns were quantified and visualized using the built-in CellChat functions, with the understanding that these measures reflect relative communication tendencies and may be influenced by cell-type abundance. Comparative analysis of signaling pathways between AAA and normal groups was performed to identify pathways showing condition-associated differences. The CCL signaling pathway was selected for focused analysis based on differential gene expression and pathway enrichment results, given its established relevance to inflammatory processes in AAA.

### 2.17 Bulk RNA-seq data analysis

The expression matrix data of two AAA-related bulk RNA-seq datasets (GSE183464 and GSE269845) were downloaded from the GEO database and processed independently using identical analysis pipelines. All analyses were performed in R (version 4.5.1). For each dataset, gene re-annotation and removal of low-expression genes were conducted prior to downstream analyses. Exploratory PCA was performed separately for each dataset for quality assessment. Differential expression analysis was then performed separately within each dataset using the limma-trend pipeline implemented in the limma (version 3.65.4) and edgeR (version 4.7.3) packages with disease status as the primary comparison (48, 49). Differentially expressed genes were visualized using volcano plots. Genes that were consistently upregulated or downregulated in both datasets were considered AAA-associated differentially expressed genes. Functional enrichment analyses, including GO and KEGG enrichment based on the overlapping DEGs, as well as GSEA based on the logFC values of all genes, were performed using the clusterProfiler package (version 4.17.0) (50).

### 2.18 Immune cell deconvolution using CIBERSORTx

Immune cell composition in bulk RNA-seq samples was estimated using the CIBERSORTx method (51), a computational framework for digital cytometry based on gene expression signatures. Normalized gene expression matrices were uploaded to the CIBERSORTx web portal (https://cibersortx.stanford.edu/index.php), and the LM22 signature matrix was used to quantify the relative fractions of 22 immune cell types. Statistical significance of each cell-type proportion was assessed using 1000 permutations, and samples with *P* < 0.05 were considered reliable for downstream analysis. Fractional abundances were further analyzed and visualized using R to compare immune cell composition across groups or conditions.

### 2.19 ScRNA-seq phenotype association using SCISSOR

Phenotype-associated single-cell populations were identified using the SCISSOR algorithm by the R package Scissor (version 2.0.0) (52), which integrates single-cell RNA-seq data with bulk phenotypic information (AAA and normal group). Each cell was assigned a score reflecting its association with the phenotype, and cells with significant positive or negative associations were selected for downstream analyses. Associated subpopulations were visualized using UMAP and heatmaps to highlight their distribution within the single-cell landscape.

### 2.20 Flow cytometry

Cells were centrifuged at 300 × g for 5 minutes at 4°C. The resulting cell pellet was resuspended in a buffer containing 0.5% bovine serum albumin. Cell viability and count were determined using trypan blue exclusion and an automated cell counter (Countess, Life Technologies, USA). Anti-CD16/CD32 antibody (BioLegend, 101302, USA) was added and incubated at room temperature for 10 minutes to minimize non-specific binding. Subsequently, cells were stained with fluorochrome-conjugated antibodies: FITC anti-mouse CD3 (BioLegend, 100203, USA), PE anti-mouse F4/80 (eBioscience, 12-4801-82, USA), and FITC anti-mouse CD19 (BioLegend, 115507, USA). Data were analyzed using FlowJo software.

### 2.21 Analysis of the UK biobank database

The UK Biobank received ethical approval from the North West Multi-Centre Research Ethics Committee (REC reference: 16/NW/0274). All participants provided informed consent to participate. The present analyses were conducted under UK Biobank application number 151350.

From a total of 502,128 participants in the UK Biobank, we excluded 450,068 individuals with missing data on the CCL20 protein level. After further excluding those with a baseline abdominal aortic aneurysm, the final analytical cohort comprised 52,017 individuals. This cohort was used to assess the association between circulating CCL20 levels at baseline and the risk of incident AAA. The baseline CCL20 levels were compared between participants who developed incident AAA and those who did not using a t-test. Multivariable Cox regression models were used to estimate hazard ratios (HR) and 95% confidence intervals (CI) for the association between circulating CCL20 level and risk of incident AAA. Multivariable-adjusted model was adjusted for age, sex, ethnicity, educational attainment, socioeconomic deprivation, body mass index, smoking status, alcohol drinking, healthy diet, leisure time physical activity, diabetes, hypertension, and hypercholesterolemia.

### 2.22 Statistical analysis

Statistical analyses were performed using GraphPad Prism 8.0 and R 4.5.1 in this study. *In vitro* and *in vivo* results are presented as mean ± standard error of the mean. Student’s t-test (normal data) or Wilcoxon test (non-normal data) was performed to compare the means between two groups. Multiple group comparisons were conducted by one-way or two-way analysis of variance (ANOVA). Correlation analysis was performed using the Spearman method by the R package ggstatsplot (version 0.13.1). Statistical graphs were visualized by GraphPad Prism 9.5 or the R package ggplot2 (version 3.5.2). *P* < 0.05 was considered significant. Details of the statistical analysis were mentioned in each Figure legend.

## 3 Results

### 3.1 Immune cell infiltration and structural cell depletion in AAA

**Figure 1:**
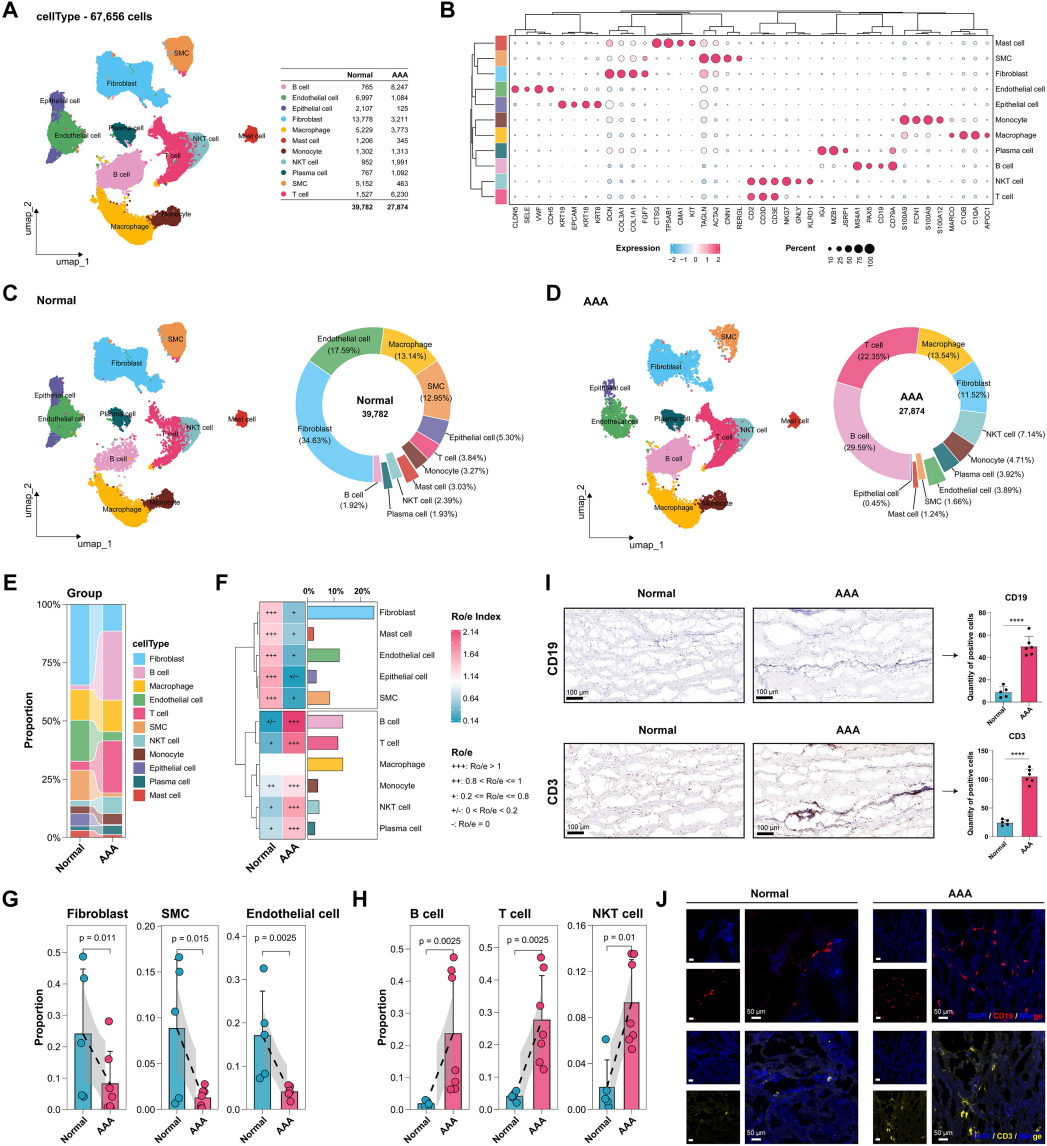
Immune Infiltration and Structural Cells Depletion in AAA. **(A)** Uniform manifold approximation and projection (UMAP) map of 11 annotated cell clusters of single-cell data (all cells). **(B)** Expression of the marker genes in each major cell type. **(C, D)** UMAP and cell composition of the normal and AAA groups. **(E)** The proportion of altered 11 cell types between the normal and AAA groups. **(F)** Ratio of observed to expected (Ro/e) analysis for cell types in different groups with cell proportion bar plot visualized at right; **(G, H)** The statistics test of the proportion of altered structural cell types and immune cell types in each sample. **(I)** Representative images of IHC of B cells (CD19) and T cells (CD3) in the the normal (n=5) and AAA group (n=6). Scale bar: 100 μm. **(J)** Representative images of IF staining of B cells (CD19, red) and T cells (CD3, yellow) in the the normal (n=5) and AAA group(n=6); nuclei were stained with DAPI (blue). Scale bar: 50 μm. ****P < 0.0001.

To examine the transcriptional landscape of the human abdominal aorta and determine cellular alterations in AAA tissues, two single-cell RNA-seq datasets (GSE166676: 2 normal, 4 AAA samples; GSE226492: 3 normal, 3 AAA samples) were downloaded from the Gene Expression Omnibus (GEO) database. After quality control and batch effects correction, 67,656 cells were obtained (39,782 normal, 27,874 AAA; **Supplementary Figures 1A–C**). All of the cells were categorized into 16 clusters (**Supplementary Figures 1D–F**). Based on cellular marker genes, cells were clustered and further annotated into 11 distinct main types (**Figures 1A, B**).

To detect the variability in the proportions of different cell types across groups, we calculated the proportions of all cells from the two groups. Compared with the normal group, the proportion of structural cells was substantially decreased in AAA tissues, including fibroblasts (from 34.63% to 11.52%), endothelial cells (from 17.59% to 3.89%), and SMCs (from 12.95% to 1.66%) (**Figures 1C–E, G**). Conversely, immune cells such as T cells (from 3.84% to 22.35%), B cells (from 1.92% to 29.59%), and NKT cells (from 2.39% to 7.14%) were significantly enriched (**Figures 1C–E, H**), indicating substantial immune cell infiltration and immune microenvironment remodeling in AAA, while the proportion of macrophages showed minimal change (from 13.14% to 13.54%) (**Figures 1C–E**).

To further evaluate the tissue distribution preferences of different cell types, we performed Ro/e preference analysis. The results demonstrated that structural cells (fibroblasts, endothelial cells, and SMCs) were less prevalent in the AAA group compared to the normal group (**Figure 1F**), whereas immune cells (T cells, B cells, and NKT cells) exhibited preferential enrichment in AAA tissues (**Figure 1F**). IHC and IF staining of human aortic tissues further confirmed the enrichment of T cells and B cells (**Figures 1I, J**; **Supplementary Figures 1G, H**). Consistent with prior research (53), findings revealed a profound cellular imbalance in AAA tissues, characterized by extensive immune cell infiltration (T cells and B cells) and loss of structural cells (fibroblasts, endothelial cells, and SMCs).

### 3.2 Inflammatory and immune responses were activated in AAA

**Figure 2:**
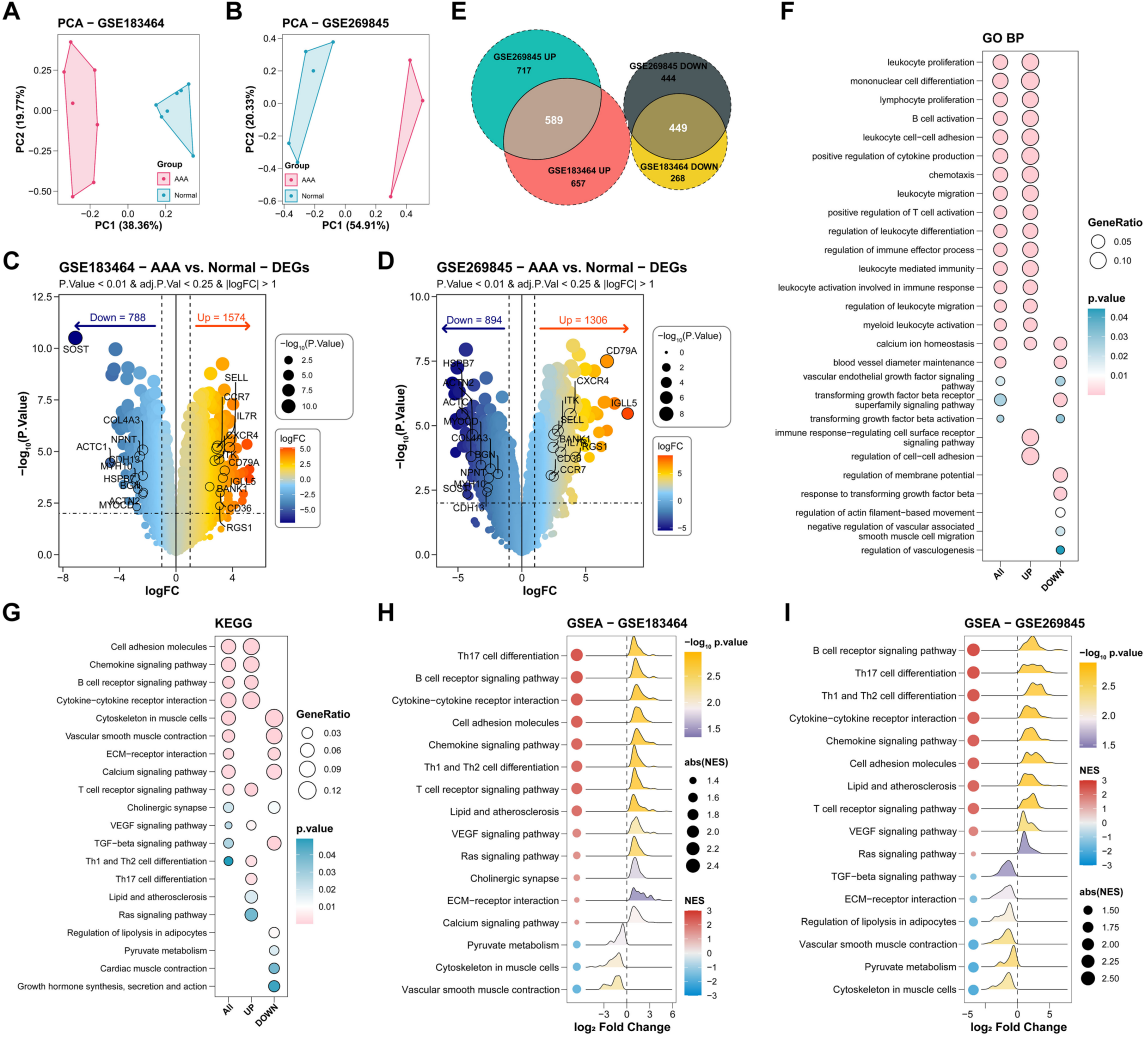
Immune-Inflammatory Activation and Vascular Structural Disruption in AAA. **(A, B)** PCA plot of the bulk RNA-seq datasets GSE183464 and GSE269845. **(C, D)** The volcano plot of DEGs in the normal and AAA groups. **(E)** Venn diagram of co-expressing upregulated and downregulated DEGs in the two datasets. **(F)** Bubble plot of GO BP enrichment results for DEGs. **(G)** Bubble plot of enriched KEGG pathways for DEGs. **(H, I)** GSEA results based on KEGG identified activated and suppressed pathways in the normal and AAA groups. Categories with red dots (NES > 0) showed the activated pathways in AAA group, while categories with blue dots (NES < 0) showed the activated pathways in the normal group.

To investigate the transcriptomic signatures between AAA and normal tissues, two human bulk RNA-seq datasets (GSE183464 and GSE269845) were downloaded from the GEO database. Results of Principal component analysis (PCA) revealed significant transcriptomic heterogeneity between the groups (**Figures 2A, B**). We then identified differentially expressed genes (DEGs) using the threshold described in the Methods section, comparing the AAA and normal groups in both datasets. Upregulated genes *(e.g., C-C motif chemokine receptor 7 (CCR7), C-X-C motif chemokine receptor 4 (CXCR4), Cluster of Differentiation 79A (CD79A)* were associated with immune responses, immune cell activation, and cell communication, whereas downregulated genes (e.g., *Actin Alpha Cardiac Muscle 1 (ACTC1), Collagen, type IV, alpha 3 chain (COL4A3), Actinin, alpha 2 (ACTN2)* were linked to vascular structure and cytoskeleton organization (**Figures 2C, D**). A Venn diagram showed that 589 genes were consistently upregulated and 449 genes were consistently downregulated in the two datasets (**Figure 2E**).

Gene Ontology (GO) and Kyoto Encyclopedia of Genes and Genomes (KEGG) pathway enrichment indicated upregulated genes were enriched in inflammatory processes, immune activation, and cytokine chemotaxis, while downregulated genes related to extracellular matrix degradation and smooth muscle contraction (**Figures 2F, G**; **Supplementary Figure 2**). Gene set enrichment analysis (GSEA) confirmed immune-related pathways (e.g., B/T cell receptor signaling, cytokine-cytokine receptor interaction, chemokine signaling, and lipid metabolism) were significantly active in AAA; whereas pathways related to aortic structure (e.g., extracellular matrix-receptor interaction, SMC cytoskeleton, and vascular smooth muscle contraction) were suppressed (**Figures 2H, I**). These results demonstrated that AAA tissues exhibited enhanced inflammatory and immune responses accompanied by the disruption of vascular wall integrity.

### 3.3 Macrophage polarization influenced the progression of AAA

**Figure 3:**
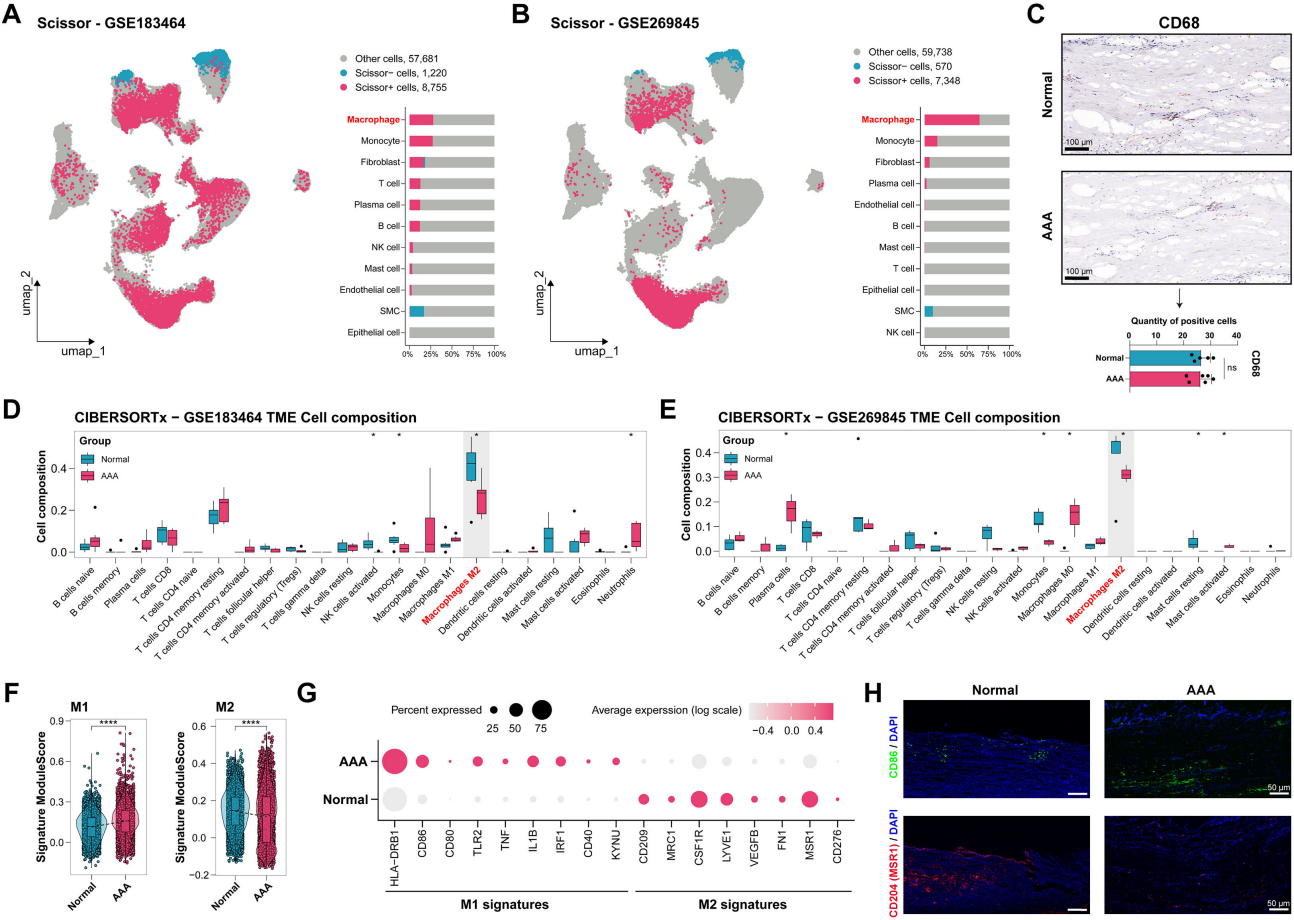
Macrophage Polarization Correlates with AAA Progression. **(A, B)** Results of AAA-related cells identification from single-cell datasets via Scissor algorithm by the bulk RNA-seq datasets GSE1834645 and GSE269845, respectively. **(C)** Representative images of IHC staining of macrophage cells (CD68) in the the normal (n=5) and AAA group(n=6). Scale bar: 100 μm. **(D, E)** CIBERSORTx was performed and calculated the relative composition of immune cell subsets between the normal and AAA group. **(F)** Signature Module Score of M1-like and M2-like macrophages in the normal and AAA groups. **(G)** Expression level of M1-like and M2-like macrophage signature genes in single-cell data in the normal and AAA groups. **(H)** Representative images of IF of M1-like macrophages marker (CD86, green) and M2-like macrophages marker (CD204, red) in the the normal (n=5) and AAA group (n=6); nuclei were stained with DAPI (blue), scale bar, 50 μm. *P < 0.05, **P < 0.01, ****P < 0.0001.

To identify cell types associated with the progression of AAA and further explore their biological characteristics, the Scissor algorithm was performed using the datasets GSE183464 and GSE269845, respectively, and a higher proportion of Scissor+ cells was obtained in the AAA group (**Supplementary Figures 3A, B**). In terms of relative proportions, there are more Scissor+ cells in macrophages, and SMC exhibited the highest proportion of Scissor- cells (**Figures 3A, B**), indicating that macrophages play a critical role in AAA pathogenesis. However, single-cell data analysis (**Figure 1E**) and IHC staining (**Figure 3C**) revealed no significant change in the proportion of macrophages between the AAA and normal groups.

CIBERSORTx was performed to investigate the difference in the composition of immune cell subsets between the AAA and normal groups, and the relative proportions of immune cell infiltration were calculated (**Supplementary Figures 3C, D**). Compared with the normal group, the AAA group showed a significant decrease in NK cells activated, monocytes, and M2 macrophages in GSE183464 (**Figure 3D**). In GSE269845, the AAA group exhibited a significant decrease in macrophages M0, and macrophages M2 (**Figure 3E**). The results demonstrated that the proportion of M2 macrophages was statistically significantly reduced in the AAA group of two datasets.

The single-cell datasets revealed that the M1-like macrophage signature module score was upregulated in the AAA group, while the normal group exhibited a higher M2-like macrophage module score (**Figure 3F**). Furthermore, macrophages in the AAA group expressed elevated M1-like macrophage signatures, while the normal group exhibited higher expression of M2-like macrophage signatures (**Figure 3G**). Additionally, Cluster of Differentiation 204 (CD204) (MSR1) was highly expressed in healthy human aortas, and a higher expression level of Cluster of Differentiation 86 (CD86) was observed in the AAA tissues (**Figure 3H**; **Supplementary Figures 3E, F**). We further characterized macrophage polarization through multiplex immunofluorescence staining by identifying additional M1/M2 markers to provide a more comprehensive assessment of macrophage polarization. In human samples, macrophages with pro-inflammatory M1-like features were identified as CD68^+^CD86^+^iNOS^+^, and macrophages with anti-inflammatory M2-like features were identified as CD68^+^CD163^+^CD206^+^, reflecting their functional polarization tendencies (**Supplementary Figures 3G, H**). Our results show that macrophages in AAA samples exhibited a stronger M1-like functional profile, indicating M1 polarization, while in normal samples, macrophages displayed higher expression of M2 markers, including CD68^+^CD163^+^CD206^+^. These findings indicated that the proportion of macrophages showed little change, while the imbalance between M1-like and M2-like macrophage polarization might influence the AAA progression.

### 3.4 Macrophages exhibited altered gene expression accompanied by abundant secretion of CCL20 in AAA

**Figure 4:**
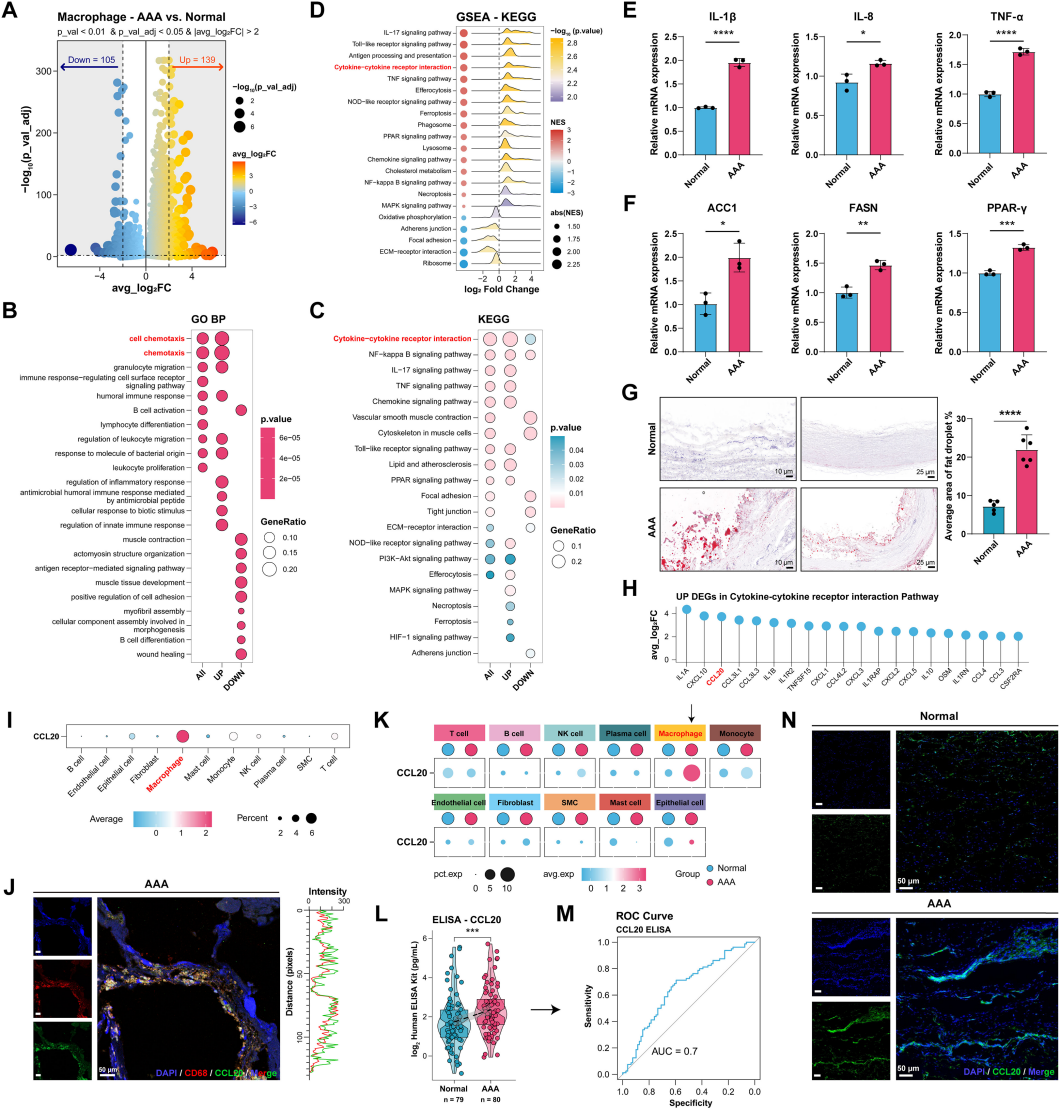
Macrophages Exhibit Altered Gene Expression and Secrete Abundant CCL20. **(A)** The volcano plot of DEGs of macrophages in the normal and AAA groups in GSE166676 and GSE226492. **(B)** Bubble plot of GO BP enrichment results for DEGs. **(C)** Bubble plot of enriched KEGG pathways for DEGs. **(D)** GSEA results based on KEGG identified activated and suppressed pathways of macrophages in the normal and AAA groups. Categories with red dots (NES > 0) showed the activated pathways in AAA group, while categories with blue dots (NES < 0) showed the activated pathways in the normal group. **(E)** The mRNA expression of IL-1beta, IL-8, TNF-α in normal and AAA tissues from human. **(F)** The mRNA expression of ACC1, FASN, and PPAR-γ in human Normal and AAA groups. **(G)** Representative images of Oil red O staining of the normal (n=5) and AAA group (n=6). Scale bar:10, 25μm. **(H)** All up DEGs in the Cytokine-Cytokine receptor interaction pathway. **(I)** CCL20 expression in 11 cell types. **(J)** Representative images of IF co-localization of CCL20 (green) and macrophages (CD68, red). Nuclei were stained with DAPI (blue), scale bar, 50 μm. The synchronized fluctuations of CCL20 (green) and macrophages (CD68, red) curves indicated co-localization at these discrete sites. **(K)** CCL20 expression in 11 cell types between the normal group and AAA groups. **(L)** ELISA test of CCL20 in the normal group (n=79) and AAA group (n=80). **(M)** ROC curve comparing models for AAA diagnosis. The model achieved an area under the curve (AUC) of 0.7. **(N)** Representative images of IF of CCL20 (green) expression in the the normal (n=5) and AAA group (n=6). Nuclei were stained with DAPI (blue), scale bar, 50 μm. *P < 0.05, **P < 0.01, ***P < 0.001, and ****P < 0.0001.

Macrophages have been shown to play a critical pathophysiological role in AAA progression (8, 54). After identifying the imbalance of macrophage polarization between the normal and AAA groups, we investigated the gene expression in macrophages. DEG analysis was performed using the single-cell datasets on macrophages between the AAA and normal groups. Compared to the normal group, 139 upregulated and 105 downregulated DEGs were obtained in AAA macrophages (**Figure 4A**). GO term and KEGG pathway enrichment analysis demonstrated that the upregulated genes were significantly associated with biological processes, including chemotaxis, cell chemotaxis, immune and inflammatory response, and pathways including *Tumor Necrosis Factor (TNF)* signaling pathway, *Peroxisome Proliferator-Activated Receptor (PPAR)* signaling pathway, chemokine signaling pathway, cytokine-cytokine receptor interaction pathway, and so on (**Figures 4B, C**). GSEA further confirmed that these processes and pathways were activated in the AAA group (**Figure 4D**). RT-qPCR was performed to validate these findings in human AAA tissues. The results showed that expression of genes related to immune response and chemotaxis (*Interleukin-1 β (IL-1β), Interleukin-8 (IL-8), and TNF-α*) was elevated (**Figure 4E**). Additionally, the expression levels of lipid metabolism-related markers (*Acetyl-CoA Carboxylase 1 (ACC1)*, *Fatty Acid Synthase (FASN)*, and *PPAR-γ*) were elevated (**Figure 4F**). Oil Red O staining also revealed notable lipid deposition in the aneurysm (**Figure 4G**). These results demonstrated inflammatory and lipid accumulation features in macrophages of AAA.

Given the significant enrichment observed (**Figures 4B, C**), we investigated the cytokine-cytokine receptor interaction pathway and ranked the average log_2_FC values of the associated upregulated DEGs (**Figure 4H**, **Supplementary Figure 4D**). Among these, Interleukin-1 α (IL-1α), C-X-C motif chemokine ligand 10 (CXCL10), and CCL20 were the top three elevated factors in macrophages from AAA. single-cell datasets revealed that CCL20 was primarily and highly expressed in macrophages across all 11 cell types analyzed (**Figure 4I**). IF co-localization further confirmed that CCL20 is predominantly present in macrophages (**Figure 4J**). Compared to the normal group, CCL20 expression in macrophages was significantly elevated in AAA tissues (**Figure 4K**), which was also evident in the GSE183464 and GSE269845 datasets (**Supplementary Figure 4A**).

Considering the secretory nature of CCL20 and its previously validated diagnostic efficacy as a biomarker (55), serum samples were collected from 80 AAA patients and 79 healthy controls for ELISA analysis (**Supplementary Table 2**). The results indicated significantly higher CCL20 levels in AAA patients compared to healthy controls (**Figure 4L**), with an AUC value of 0.7 (**Figure 4M**).

To make an external validation, we analyzed the data from the UK Biobank. In the cohort of 52,017 participants, 329 incident AAA cases were identified during a median follow-up of 13.59 years. The demographic characteristics of participants were shown in **Table 1**. Multivariable Cox regression results showed that a per 1-SD increase in circulating CCL20 level was associated with increased risk of AAA. Compared to participants with the lowest quartiles (Q1) of CCL20, those with the highest quartiles (Q4) had a 44% increased risk of incident AAA (HR: 1.44; 95% CI: 1.02, 2.03; *P* = 0.040) (**Table 2**, **Supplementary Figure 4C**). Additionally, CCL20 expression was increased in human AAA tissues relative to normal abdominal aortic tissues (**Figure 4N**, **Supplementary Figure 4B**). These findings suggest that CCL20 is primarily secreted by macrophages in AAA, with elevated levels in both tissues and blood, potentially implicating it in AAA progression.

**Table 1:** Demographic characteristics of participants from UK Biobank database.

Characteristic	Total population	Participants without AAA	Participants with AAA	*P*-value
N	52,017	51,688	329	
Age, years	56.79 ± 8.21	56.75 ± 8.21	63.50 ± 5.49	<0.001
Male, n (%)	23965 (46.07)	23690 (45.83)	275 (83.59)	<0.001
Ethnicity, n (%)				0.124
White	48524 (93.28)	48205 (93.26)	319 (96.96)	
Mixed background	343 (0.66)	343 (0.66)	0 (0.00)	
South Asian	959 (1.84)	959 (1.86)	0 (0.00)	
Black	1183 (2.27)	1178 (2.28)	5 (1.52)	
Chinese	145 (0.28)	144 (0.28)	1 (0.30)	
Other	608 (1.17)	605 (1.17)	3 (0.91)	
Missing	255 (0.49)	254 (0.49)	1 (0.30)	
Educational level, n (%)				<0.001
No relevant qualifications	16605 (31.92)	16546 (32.01)	59 (17.93)	
CSEs or equivalent	5691 (10.94)	5668 (10.97)	23 (6.99)	
O levels, GCSEs, or equivalent	10735 (20.64)	10666 (20.64)	69 (20.97)	
A level, AS levels, or equivalent	2773 (5.33)	2761 (5.34)	12 (3.65)	
College or university degree	6174 (11.87)	6111 (11.82)	63 (19.15)	
Others	9155 (17.60)	9055 (17.52)	100 (30.40)	
Missing	884 (1.70)	881 (1.70)	3 (0.91)	
Townsend deprivation index, n (%)				0.711
Q1	10288 (19.78)	10224 (19.80)	64 (19.45)	
Q2	10233 (19.67)	10169 (19.70)	64 (19.45)	
Q3	9841 (18.92)	9774 (18.93)	67 (20.36)	
Q4	10568 (20.32)	10510 (20.36)	58 (17.63)	
Q5	11024 (21.19)	10948 (21.21)	76 (23.10)	
Missing	63 (0.12)			
Body mass index, n (%)				<0.001
kg/m^2^	268 (0.52)	267 (0.52)	1 (0.30)	
18.5 to <25 kg/m^2^	16549 (31.81)	16479 (32.04)	70 (21.28)	
25 to <30 kg/m^2^	22222 (42.72)	22068 (42.91)	154 (46.81)	
≥30 kg/m^2^	12724 (24.46)	12620 (24.54)	104 (31.61)	
Missing	254 (0.49)			
Smoking status, n (%)				<0.001
Never	28163 (54.14)	28102 (54.37)	61 (18.54)	
Past	18121 (34.84)	17966 (34.76)	155 (47.11)	
Current	5479 (10.53)	5367 (10.38)	112 (34.04)	
Missing	254 (0.49)	253 (0.49)	1 (0.30)	
Alcohol drinking, n (%)				0.997
Never	2456 (4.72)	2441 (4.72)	15 (4.56)	
Past	2019 (3.88)	2006 (3.88)	13 (3.95)	
Current	47406 (91.14)	47106 (91.14)	300 (91.19)	
Missing	136 (0.26)	135 (0.26)	1 (0.30)	
Leisure time physical activity, n (%)				0.037
MET/min/weeks	18200 (34.99)	18096 (35.01)	104 (31.61)	
500 to <1000 MET/min/weeks	10689 (20.55)	10630 (20.57)	59 (17.93)	
≥1000 MET/min/weeks	18424 (35.42)	18301 (35.41)	123 (37.39)	
Missing	4704 (9.04)	4661 (9.02)	43 (13.07)	
Healthy dietary, n (%)	2356 (4.53)	2342 (4.53)	14 (4.26)	0.884
Missing	1971 (3.79)	1960 (3.79)	11 (3.34)	
Diabetes, n (%)	2950 (5.67)	2920 (5.65)	30 (9.12)	0.024
Missing	227 (0.44)	226 (0.44)	1 (0.30)	
Hypertension, n (%)	14592 (28.05)	14429 (27.92)	163 (49.54)	<0.001
Missing	61 (0.12)	61 (0.12)	0 (0.00)	
Hyperlipidemia, n (%)	6710 (12.90)	6587 (12.74)	123 (37.39)	<0.001

**Table 2:** High circulating CCL20 level predicted higher risk of AAA.

Associations between baseline circulating level and risk of AAA.				
Variable	Crude HR (95% CI)	*P*-value	Adjusted HR (95% CI) *	*P*-value
CCL20, per 1-SD increase	1.26 (1.15, 1.37)	<0.001	1.11 (1.00, 1.23)	0.047
CCL20, quartiles				
Q1	1.0		1.0	
Q2	1.30 (0.90, 1.89)	0.167	1.05 (0.72, 1.53)	0.803
Q3	2.13 (1.51, 2.99	<0.001	1.43 (1.01, 2.02)	0.044
Q4	2.47 (1.77, 3.45)	<0.001	1.44 (1.02, 2.03)	0.040

*Adjusted for age, sex, ethnicity, educational attainment, socioeconomic deprivation, body mass index, smoking status, alcohol drinking, healthy diet, leisure time physical activity, diabetes, hypertension, and hypercholesterolemia.

### 3.5 Macrophages recruited significant numbers of immune cells through the CCL20-CCR6 axis

**Figure 5:**
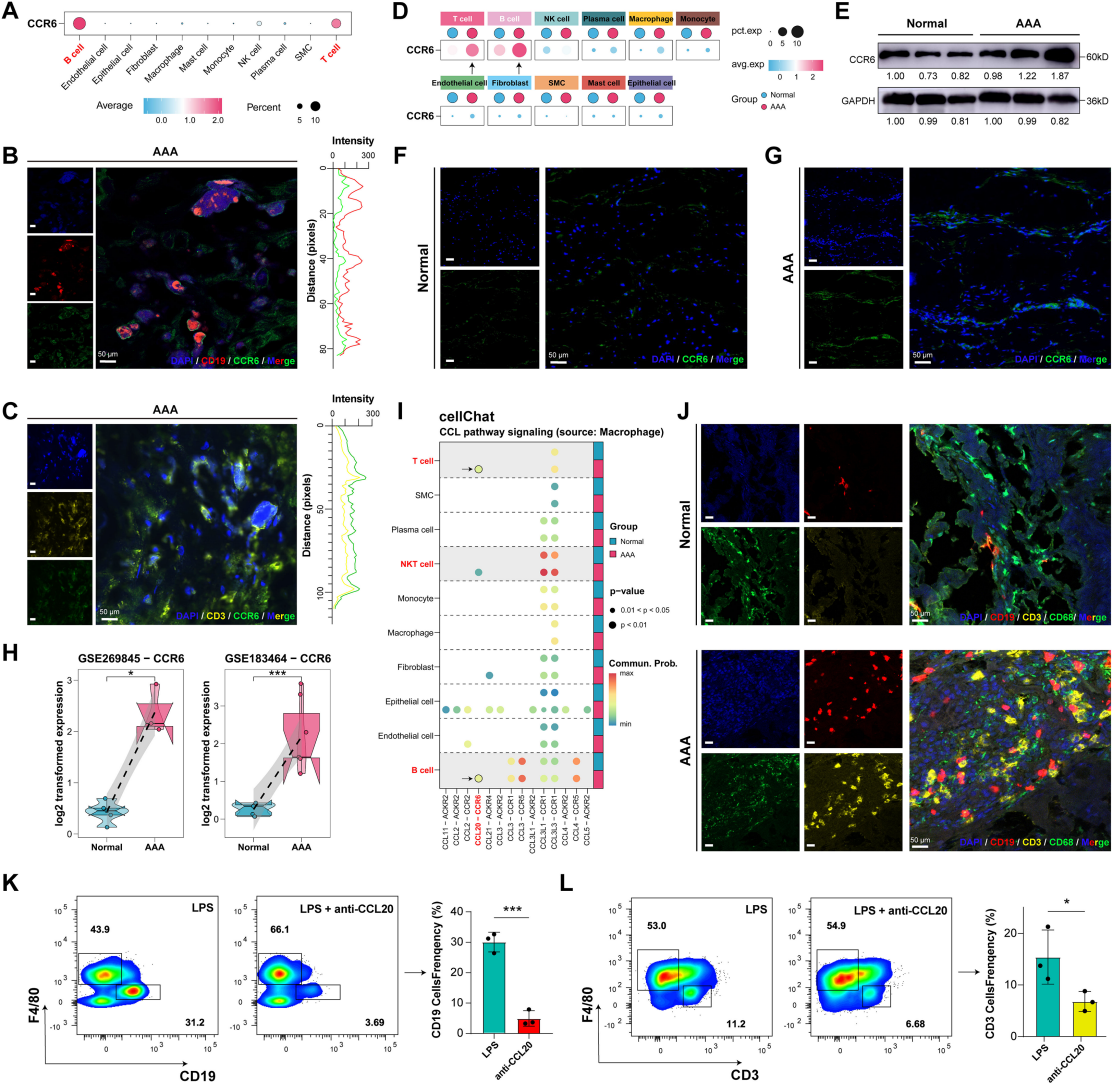
Macrophages Recruit Significant Numbers of Immune Cells through the CCL20-CCR6 Axis. **(A)** CCR6 expression in 11 cell types in GSE166676 and GSE226492. **(B)** Representative images of IF co-localization of CCR6 (green) and B cells (CD19, red). Nuclei were stained with DAPI (blue), scale bar, 50μm. **(C)** Representative images of IF co-localization of CCR6 (green) and T cells (CD3, yellow). Nuclei were stained with DAPI (blue), scale bar, 50μm. The synchronized fluctuations of CCR6 (green)and T cells (CD3, yellow) curves indicated co-localization at these discrete sites. **(D)** CCR6 expression in 11 cell types. **(E)** CCR6 protein expression in human tissues between the normal group and the AAA group. **(F, G)** Representative images of IF of CCR6 (green) expression in the the normal (n=5) and AAA group (n=6). Nuclei were stained with DAPI (blue), scale bar, 50 μm. **(H)** CCR6 mRNA expression in GSE269845 and GSE183464. **(I)** Cell Chat between macrophages and other cells in the CCL pathway signaling. **(J)** Representative images of IF of Macrophages (CD68, green), B cells (CD19, red), T cells (CD3, yellow) in the normal and AAA groups. Nuclei were stained with DAPI (blue), scale bar, 50 μm. **(K)** Flow cytometry of CD19 cells frequency in the LPS (100ng/ml) group and the LPS + CCL20 Antibody group **(L)** Flow cytometry of CD3 cells frequency in LPS (100ng/ml) group and the LPS + CCL20 Antibody group, *P < 0.05, **P < 0.01, ***P < 0.001.

As CCR6 is the sole receptor for CCL20 (33, 36, 37), we visualized its interaction relationship with CCL20 in the Cytokine-cytokine receptor interaction pathway (**Supplementary Figure 4D**). Single-cell datasets showed that CCR6 was mainly expressed in T cells and B cells (**Figure 5A**), a finding confirmed by IF co-localization in human AAA tissues (**Figures 5B, C**). Comparative analysis revealed significantly elevated CCR6 expression in T cells and B cells from AAA tissues (**Figure 5D**). Western blot and IF analyses further demonstrated higher CCR6 expression in human AAA tissues (**Figures 5E–G**; **Supplementary Figures 5A, B**). Consistently, CCR6 mRNA levels were upregulated in bulk RNA-seq datasets (**Figure 5H**). Thus, CCR6 expression was elevated in AAA tissues, primarily in T cells and B cells.

To explore potential chemokine-mediated communication involving macrophages during AAA, we performed CellChat analysis focusing on the CCL chemokine family. This analysis suggested enriched CCL-related communication patterns between macrophages and multiple immune cell types in AAA samples. In particular, inferred CCL20-CCR6-associated communication between macrophages and T cells, B cells, and NKT cells was predominantly observed in AAA tissues (**Figure 5I**). Consistent with the cellular composition revealed by single-cell datasets (**Figures 1C, D**), immunofluorescence analysis of human tissues showed no significant difference in macrophage abundance between groups, but demonstrated increased infiltration of adaptive immune cells, including T cells and B cells, in AAA tissues (**Figure 5J**). These observations, together with the inferred communication patterns, suggested a potential role for macrophage-derived CCL20-CCR6 signaling in immune cell recruitment within AAA lesions. To functionally test this hypothesis, we performed Transwell co-culture experiments using LPS-stimulated macrophages and T or B cells in the presence of a CCL20-neutralizing antibody (10 ng/ml). Flow cytometric analysis showed that blockade of the CCL20-CCR6 axis significantly reduced the migration of both T cells and B cells (**Figures 5K, L**). Collectively, these functional data support a role for macrophage-derived CCL20-CCR6 signaling in promoting immune cell migration, providing experimental validation for the communication patterns suggested by CellChat analysis.

### 3.6 Targeting the CCL20-CCR6 axis inhibited AAA progression

**Figure 6:**
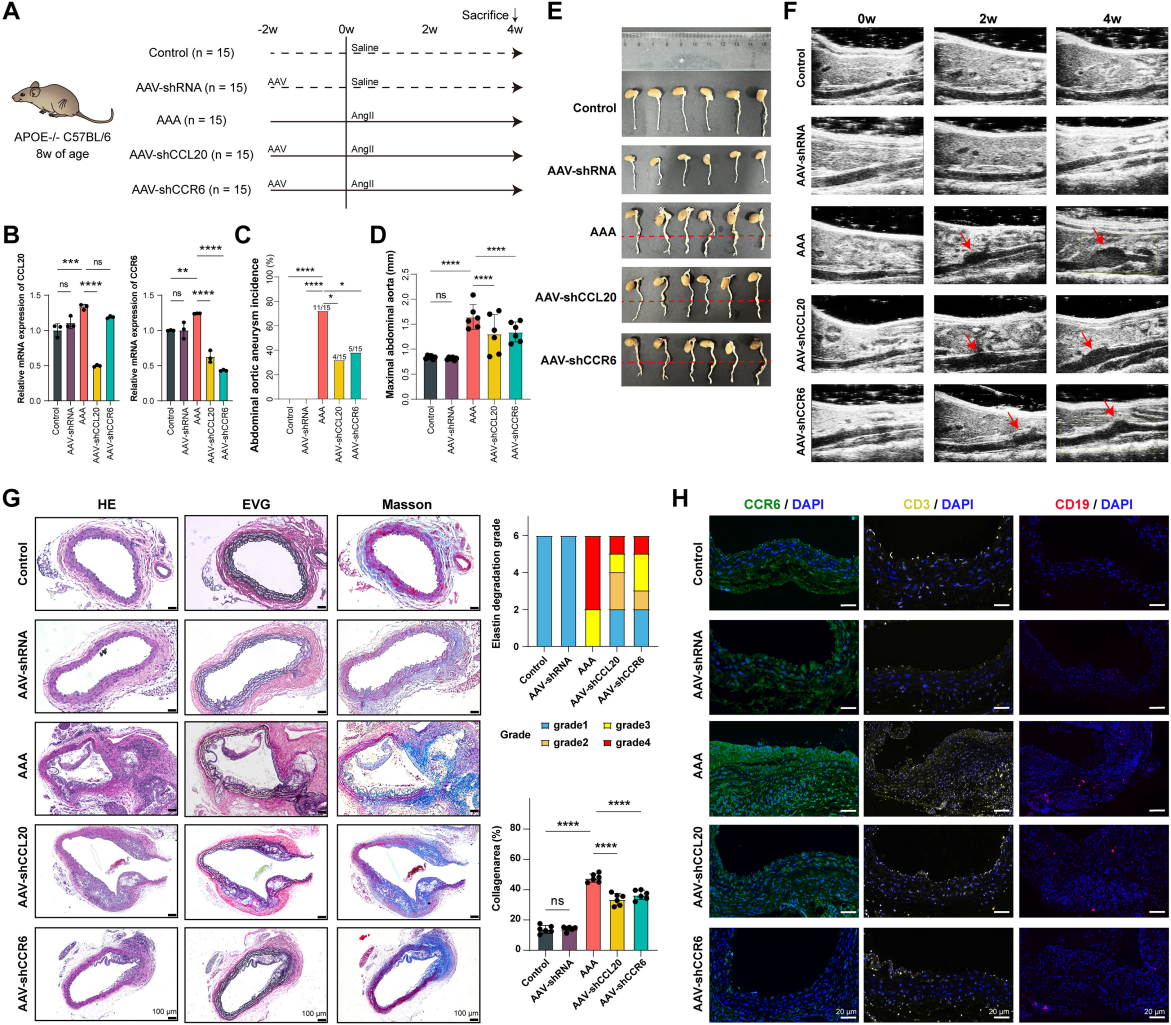
Targeting the CCL20-CCR6 Axis Suppresses AAA Progression. **(A)** Schematic representation of study design, n=15 per group. **(B)** RT-qPCR of knockdown efficiencies for CCL20 and CCR6. **(C)** The AAA incidence. **(D)** The maximal external aortic diameter (mm). **(E)** Representative images of morphology of the whole aorta of all groups. **(F)** Representative vascular Doppler ultrasound images of the aorta of all groups. (N = 15). **(G)** Representative hematoxylin and eosin (HE), Elastica van Gieson (EVG), and elastin degradation grading, Masson trichrome staining. **(H)** Representative IF images of CCR6 (green), T cells (CD3, yellow), and B cells (CD19, red) in the aorta tissues of all groups. Nuclei were stained with DAPI (blue), scale bar, 20 μm. *P < 0.05, **P < 0.01, ***P < 0.001, and ****P < 0.0001.

To validate the role of the CCL20-CCR6 axis in AAA development, we established an AAA model in *ApoE*^–/–^ mice through Ang-II infusion (1,000 ng/kg/min). Interventions were administered via tail vein injections of a specific knockdown of AAV-shCCL20/AAV-shCCR6 (**Figure 6A**).

Specifically, RT-qPCR validated the knockdown efficiencies of CCL20 and CCR6 in aortic tissues. Expression levels of both CCL20 and CCR6 were significantly higher in the AAA group compared to controls (**Figure 6B**). Knockdown of CCL20 or CCR6 markedly reduced AAA incidence (**Figure 6C**). These findings were further corroborated by vascular Doppler ultrasound imaging and measurements of maximal external aortic diameter (**Figures 6D–F**). Morphological and histological analyses demonstrated that disrupting the CCL20-CCR6 axis attenuated inflammation, elastin degradation, and collagen deposition in the aortic wall (**Figure 6G**). Additionally, IF and IHC staining revealed elevated CCR6 expression alongside abundant infiltration of T cells and B cells in AAA tissues (**Supplementary Figures 5C, D**). In contrast, knockdown of CCL20 or CCR6 diminished the accumulation of these immune cells (**Figure 6H**). By using F4/80^+^CD86^+^iNOS^+^ as representative M1-like markers and F4/80^+^CD206^+^Arg1^+^ as representative M2-like markers in multiplex immunofluorescence, we further validated the macrophage polarization status. The experimental results indicate that blockade the CCL20-CCR6 axis weakened the pro-inflammatory M1 polarization tendency and enhanced the anti-inflammatory M2 polarization tendency (**Supplementary Figure 6**). Collectively, these results indicate that targeting the CCL20-CCR6 axis reduces T cell and B cell infiltration and suppresses AAA progression.

## 4 Discussion

**Figure 7:**
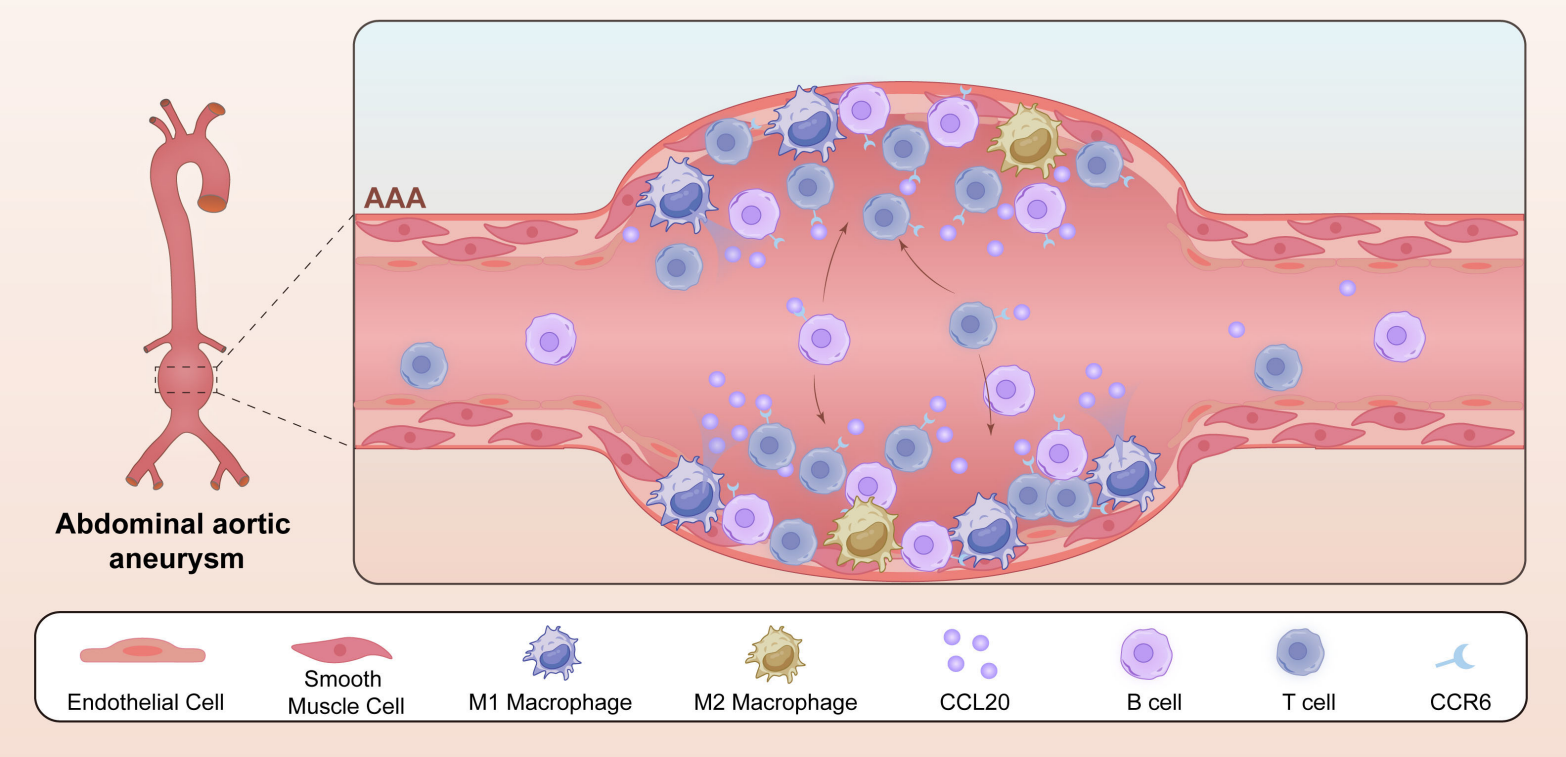
The role of CCL20-CCR6 axis in AAA formation. Macrophage polarization was imbalanced, with enriched M1-like macrophages and elevated CCL20 secretion. CCL20 promoted the recruitment of CCR6+ immune cells (T and B cells) and AAA formation.

This study employed integrated single-cell and bulk RNA sequencing, alongside experimental and external data validations, to elucidate the cellular and molecular underpinnings of AAA pathogenesis. Our analyses revealed profound immune cell infiltration (particularly T cells and B cells) and structural cell depletion (fibroblasts, endothelial cells, and SMCs) in AAA tissues, driven by imbalanced macrophage polarization favoring pro-inflammatory M1-like macrophages. This imbalance promoted CCL20 secretion, which recruits CCR6-expressing immune cells (CCR6+ T cells and CCR6+ B cells) into the aneurysmal tissue. ELISA and IF analyses confirmed significantly elevated CCL20 levels in serum and tissue samples from AAA patients. Using a knockdown model of the CCL20-CCR6 axis in AAA and an *in vitro* neutralization model of CCL20-mediated macrophage chemotaxis, we evaluated the axis’s impact on AAA. Our findings highlight the CCL20-CCR6 axis as a key regulator of AAA pathogenesis (**Figure 7**) and a potential therapeutic target.

Macrophage infiltration and polarization evolve throughout aneurysm development (8). We note that M1/M2-like macrophage signatures represent simplified polarization axes and do not capture the full spectrum of macrophage heterogeneity; therefore, these results should be interpreted as functional tendencies rather than discrete macrophage subtypes. Additionally, cytokine-cytokine receptor interaction pathways were highly activated in AAA macrophages. Our results showed no significant difference in macrophage proportions between the AAA and normal groups with the imbalance between M1-like and M2-like macrophage polarization. Macrophage polarization is a critical driver of AAA (56–59). Pro-inflammatory M1 macrophages accelerate AAA by exacerbating inflammation and secreting cytokines (22, 60, 61). In our study, M1-like macrophage polarization and altered gene expression profiles were evident in AAA macrophages, with an imbalance favoring M1-like over M2-like. IL-1α, CXCL10, and CCL20 emerged as the top three secreted chemokines, with IL-1α and CXCL10 previously implicated in AAA pathogenesis (62, 63). This study focuses on the third key chemokine, CCL20, and its exclusive receptor, CCR6, to delineate their specific roles in shaping the AAA immune microenvironment and driving AAA progression (64). The CCL20-CCR6 axis regulates immune responses in cardiovascular diseases (37), but its role in AAA remains underexplored. We observed that M1-like macrophage polarization significantly increased CCL20 expression in AAA serum and tissues. Consistent with the previous study (55), CCL20 serves as a potential AAA biomarker in large cohorts based on our and UK Biobank data. Due to challenges in obtaining normal aortic tissue, the sample size for the normal group (n=5) was relatively small. However, our study has demonstrated the pivotal role of the CCL20-CCR6 axis in AAA. Future research with larger sample sizes will be essential.

Immune cell infiltration has long been recognized as a fundamental mechanism in the chronic inflammatory progression of AAA, with chemokines playing a pivotal role (20, 65, 66). Consistent with previous findings (67), our single-cell analysis revealed a profound cellular imbalance in AAA tissues, characterized by extensive immune cell infiltration (T cells and B cells) and loss of structural cells (fibroblasts, endothelial cells, and SMCs). Ro/e analysis further demonstrated that immune cells (T cells and B cells) exhibited a significant tissue preference towards AAA tissues, suggesting a strong association with aneurysm progression risk. Given the limited donor number, Ro/e analysis was used as a descriptive tool to summarize cell-type distribution trends, and future studies with larger cohorts will be required for robust donor-level inference. CellChat analysis indicated macrophages emerged as particularly prominent in communication with T cells, B cells, and NKT cells in AAA group. CellChat-based communication analyses are inferential and hypothesis-generating, and future donor-aware modeling and functional validation will be required to further define the biological relevance of these signaling pathways.

We propose that the active CCL20-CCR6 axis provide a mechanistic framework to explain this specific recruitment of CCR6-expressing immune cells (CCR6+ T cells and CCR6+ B cells) and the ensuing inflammatory cascade. T cells represent the dominant immune cell population in AAA (25), where they can promote AAA formation and stimulate macrophages to produce pro-inflammatory mediators in AAA models like Interleukin-17 (IL-17) and TNF-α (15, 68, 69). IL-17 can stimulate SMCs and fibroblasts to secrete matrix metalloproteinases and pro-inflammatory cytokines including CCL20, thereby establishing a positive feedback loop that sustains continuous lymphocyte recruitment (70, 71). Furthermore, cytokines from other T cell subsets, such as Interferon-gamma (IFN-γ) from Th1 cells, can contribute to orchestrate extracellular matrix remodeling (68). Concurrently, infiltrating B cells influence AAA progression by secreting immunoglobulins (e.g., Immunoglobulin A&Immunoglobulin G) and cytokines like IL-6 and TNF-α, which degrade the aortic wall and exacerbate inflammatory responses (16). Depleting B cells protects mice from experimental AAA and fosters an immunosuppressive aortic environment (30). The CCL20-CCR6 axis links macrophage activation to the targeted recruitment of CCR6+ T cells and CCR6+ B cells. The secretory products of these effector cells act back on vascular wall cells and immune cells, aggravating the inflammatory response and accelerating aneurysm progression.

Inhibiting chemokine axes has proven effective in AAA animal models, such as CCL5-CCR5 or CCL2-CCR2 pathways (72–74). The CCL20-CCR6 axis is vital in regulating immune responses during chronic inflammation in AAA, underscoring the need to develop therapeutic strategies targeting the axis. Overexpressed CCL20 promotes aortic elastin degradation, inhibits M2 polarization, and accelerates AAA (34), suggesting it exacerbates inflammation and drives progression. In atherosclerosis, the deletion of CCR6 effectively ameliorates inflammatory progression and plaque size (41, 75). However, the mechanisms and efficacy of targeting CCL20-CCR6 in AAA are unclear. Our study provides initial evidence that blockade of this axis reduces aortic diameter, aneurysm incidence, and immune cells infiltration in AngII-induced ApoE^–/–^ male mice AAA model under a high-fat diet, potential gender bias in the effects should be considered. Consistent with prior studies, our AAA model successfully recapitulates the key pathological features of human AAA, including chronic inflammation and macrophage-dominated immune infiltration (11, 76). Although there are potential differences in the specific immune cell subset proportions and the disease timeline from AAA in human, the model has been used to explore the role of CCL20-CCR6 axis in AAA-related vascular lesions and immune features (34).

This study integrates bioinformatics analysis with experimental validation to explore the cellular and molecular mechanisms underlying AAA pathogenesis. Our analysis reveals a key role of CCL20 secretion, driven by an imbalance in M1-like macrophages polarization, which recruits CCR6+ immune cells (such as CCR6+ T cells and CCR6+ B cells) to promote AAA development. Our experimental framework already integrates two levels of evidence: 1) Genetic Evidence *in vivo* (AAV-shCCL20 knockdown) demonstrating the necessity of CCL20 in disease progression within the complex tissue microenvironment; 2) Pharmacological Evidence *in vitro* (CCL20-neutralizing antibody) confirming the functional consequence of blockade the CCL20-CCR6 axis. While our genetic intervention lays a crucial mechanistic groundwork, future pharmacological validation using CCL20-neutralizing antibodies or receptor antagonists *in vivo* will be an essential next step toward clinical translation.

## 5 Conclusion

Macrophage-derived CCL20 recruited CCT6+ lymphocytes and promoted AAA progression. Inhibition of the CCL20-CCR6 axis reduced the recruitment of immune cells and relieved AAA progression. CCL20 may serve as a novel diagnostic biomarker and therapeutic target for AAA.

## Data Availability

The original contributions presented in the study are included in the article/**Supplementary Material**. Further inquiries can be directed to the corresponding author/s.
