# Supplementary material for: Macrophage-derived CCL20 promotes abdominal aortic aneurysm progression via lymphocytes CCR6
Source: Front Immunol. 2026 Mar 2;17:1780720. doi: 10.3389/fimmu.2026.1780720 (PMC12989375; doi:10.3389/fimmu.2026.1780720)
Supplement: Supplementary file 1 [file DataSheet1.zip › Supplementary Material/Supplementary Material/Supplementary Figure and Table.docx]

Supplementary Material

# Supplementary Figures and Tables

## Supplementary Figures

**Supplementary Figure 1**


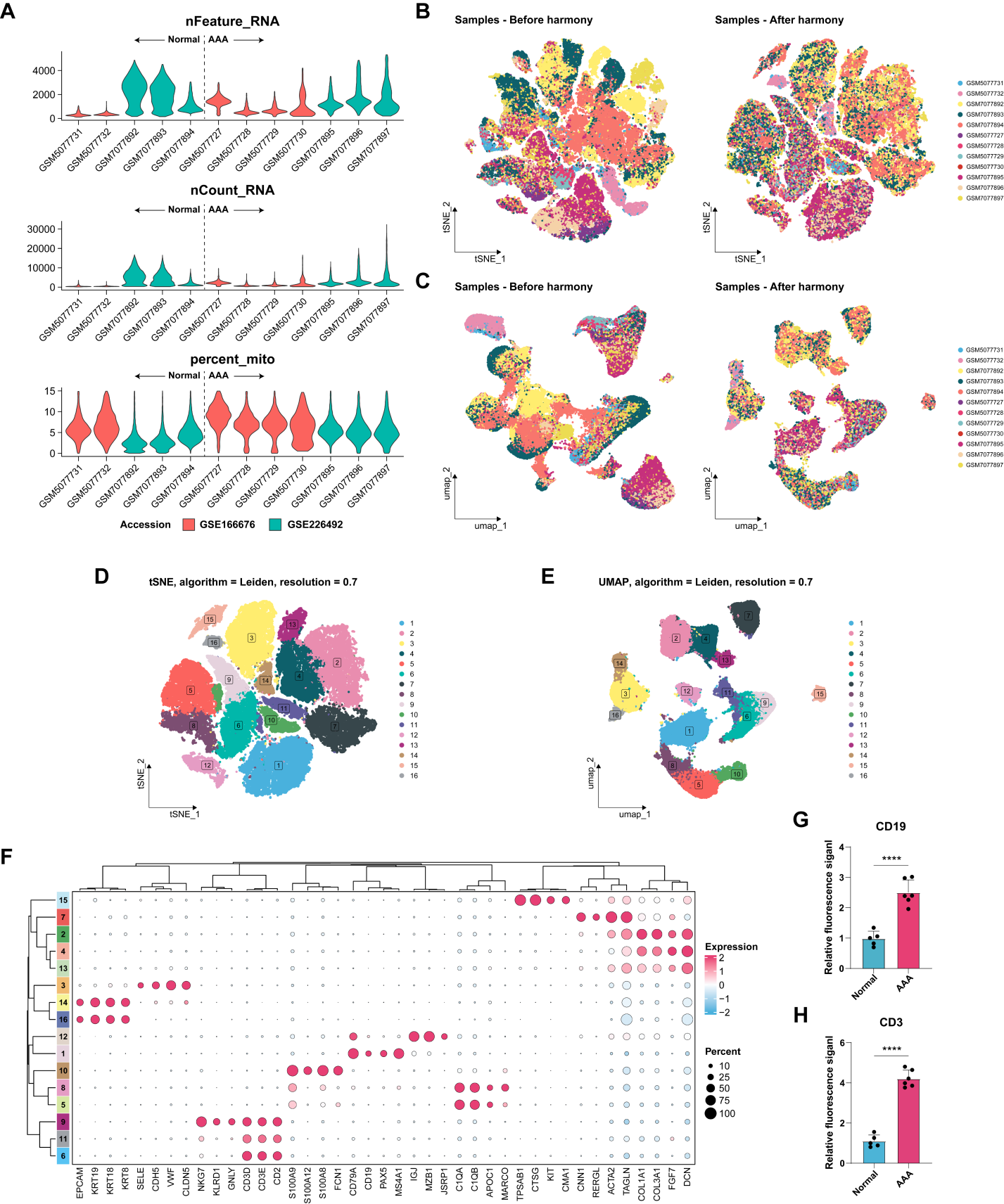


**Supplementary Figure 1 .** (A) Two single-cell RNA sequencing datasets (GSE166676: 2 normal, 4 AAA samples; GSE226492: 3 normal, 3 AAA samples) were downloaded and quality control. (B-C) Batch effect removal of two single-cell RNA sequencing datasets. (D-E) All of the cells were categorized into 16 clusters by Leiden algorithm, resolution = 0.7. (F) Expression of the marker genes in each major cell cluster. (G-H) The relative fluorescence signal level of CD19 and CD3 between the normal (n=5) and AAA groups(n=6). **P* < 0.05, ***P* < 0.01, ****P* < 0.001, and *****P* < 0.0001.

**Supplementary Figure 2**


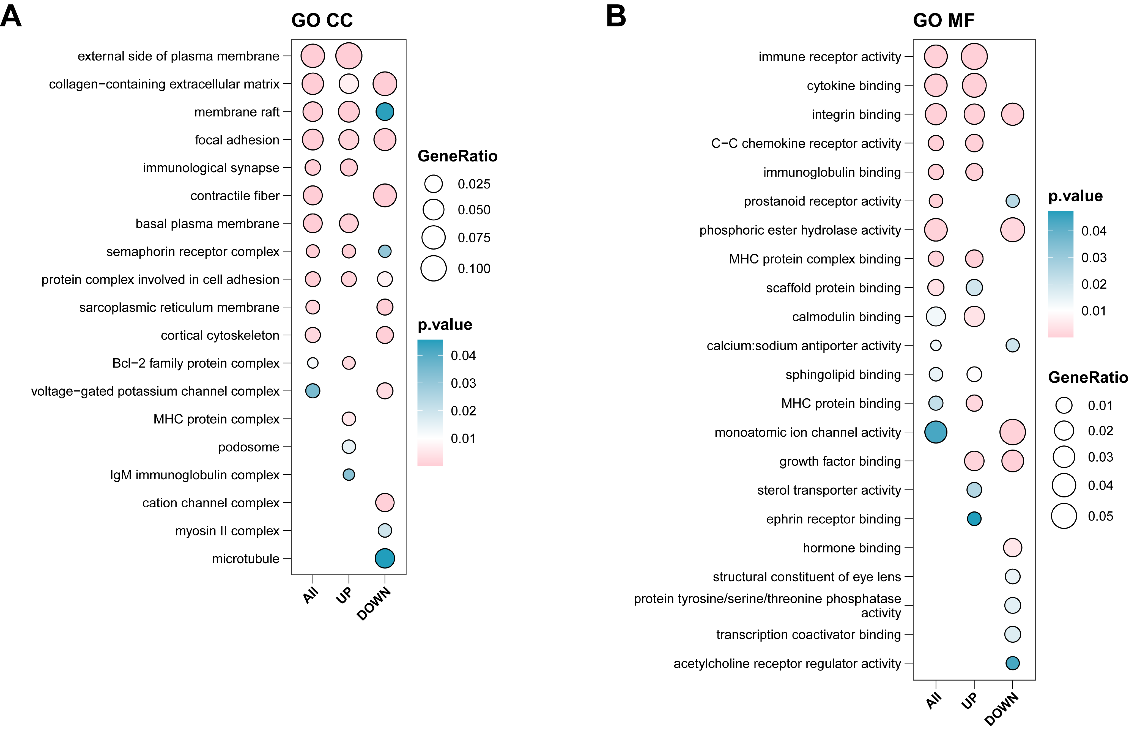


**Supplementary Figure 2.** (A-B) Bubble plot of GO CC and GO MF enrichment results for DEGs.

**Supplementary Figure 3**


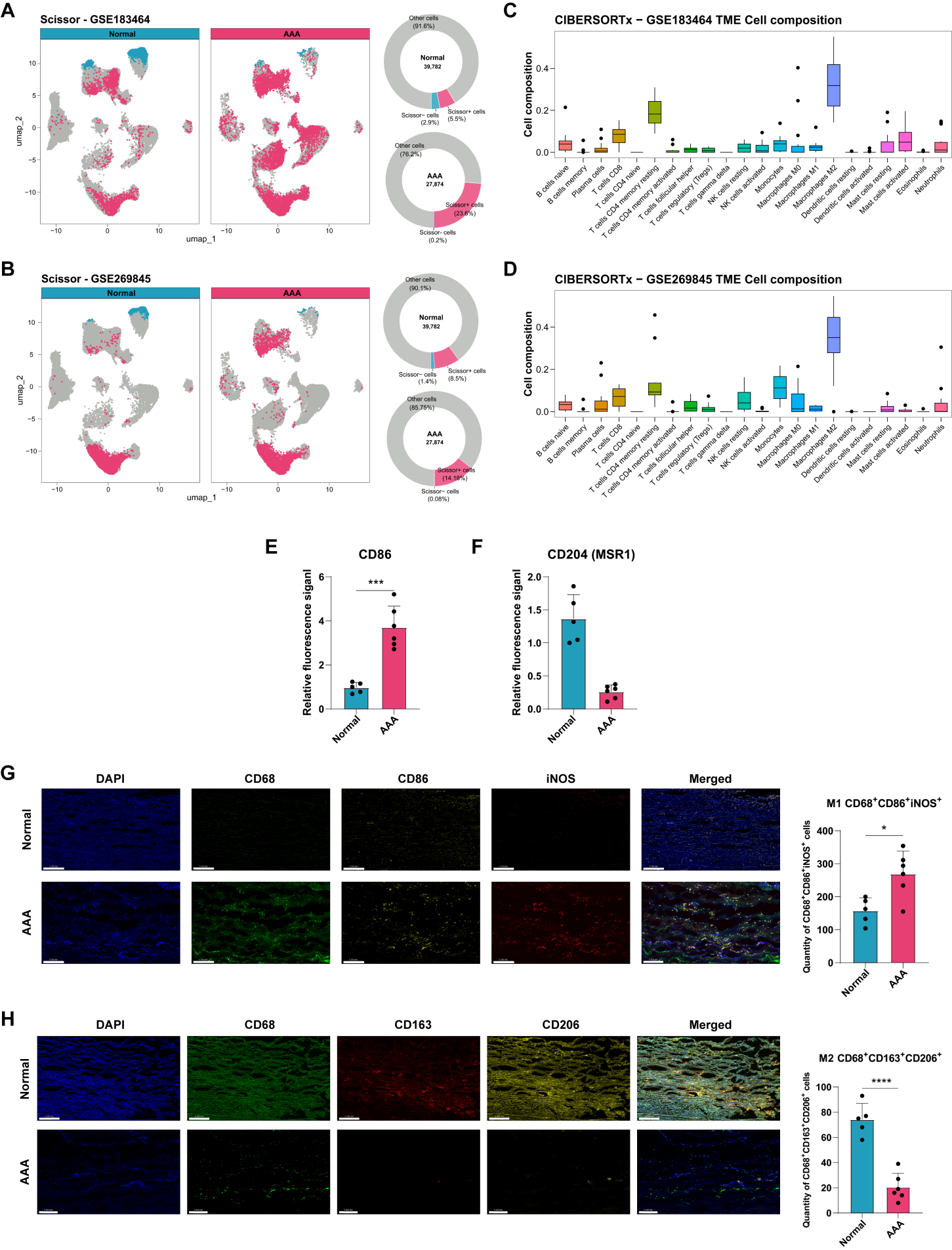


**Supplementary Figure 3.** (A-B) Results of AAA-related cells identification from single-cell datasets via Scissor algorithm by the bulk RNA-seq datasets GSE1834645 and GSE269845, respectively. (C-D) CIBERSORTx was performed and calculated the relative composition and abundance of immune cell subsets in every sample between the normal and AAA group. (E-F) The relative fluorescence signal level of CD86 and CD204 between the normal (n=5) and AAA groups(n=6). **P* < 0.05, ***P* < 0.01, ****P* < 0.001, and *****P* < 0.0001. (G-H) Representative images of IF of M1-like macrophages (CD68, green; CD86, red; iNOS, red) and M2-like macrophages (CD68,green, CD163, red; CD206, yellow); nuclei were stained with DAPI (blue), scale bar, 200mm. * *P* < 0.05, **** *P* < 0.0001.

**Supplementary Figure 4**


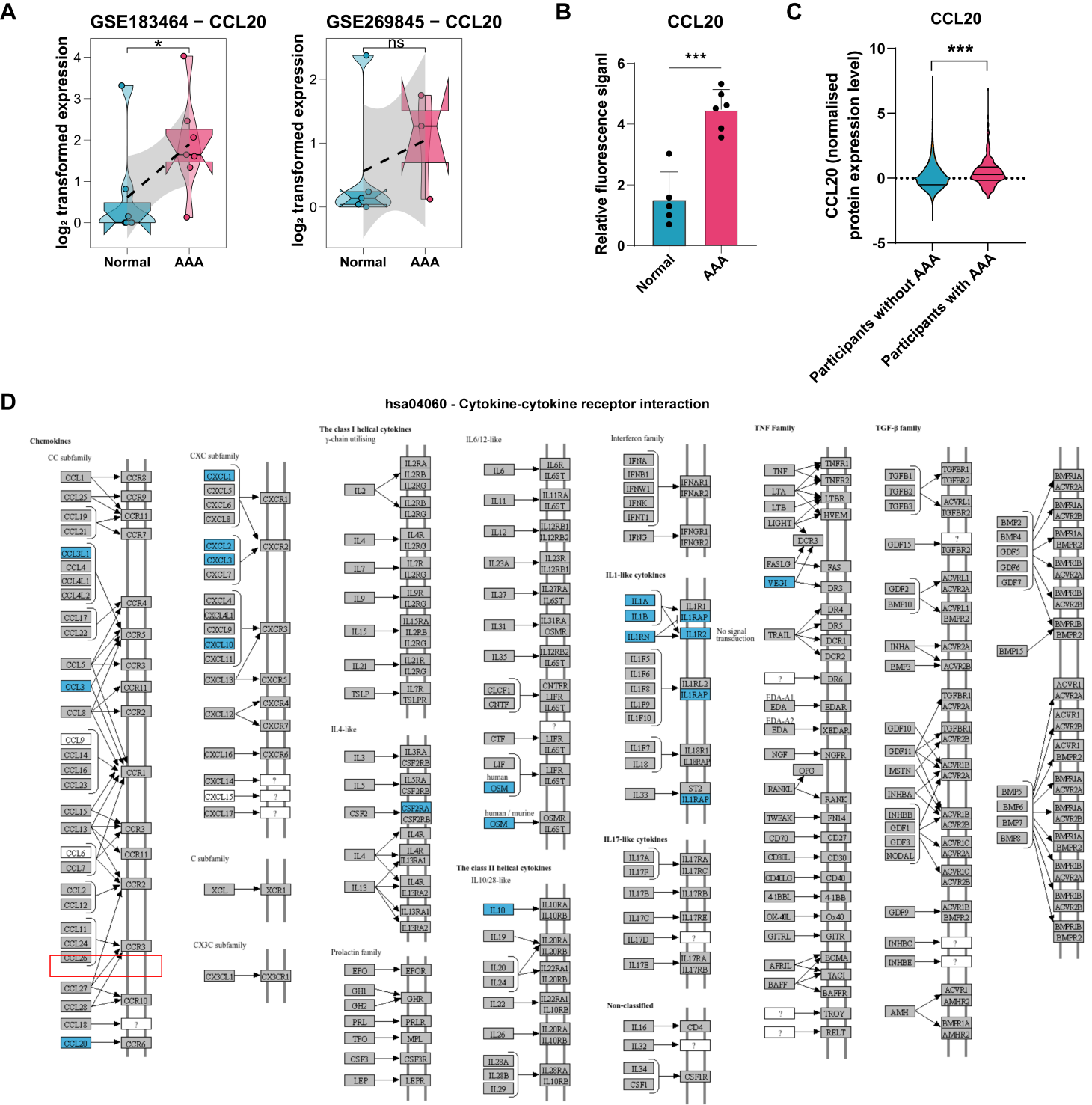


**Supplementary Figure 4.** (A) The transformed expression level of CCL20 between the Normal and AAA groups in GSE183464 and GSE269845. (B) The relative fluorescence signal level of CCL20 between the the normal (n=5) and AAA groups(n=6), **P* < 0.05, *****P* < 0.0001. (C) Comparisons of baseline CCL20 Level between participants with incident AAA cases and without AAA cases. (D) All the chemokines and its receptors of Cytokine-cytokine receptor interaction.

**Supplementary Figure 5**


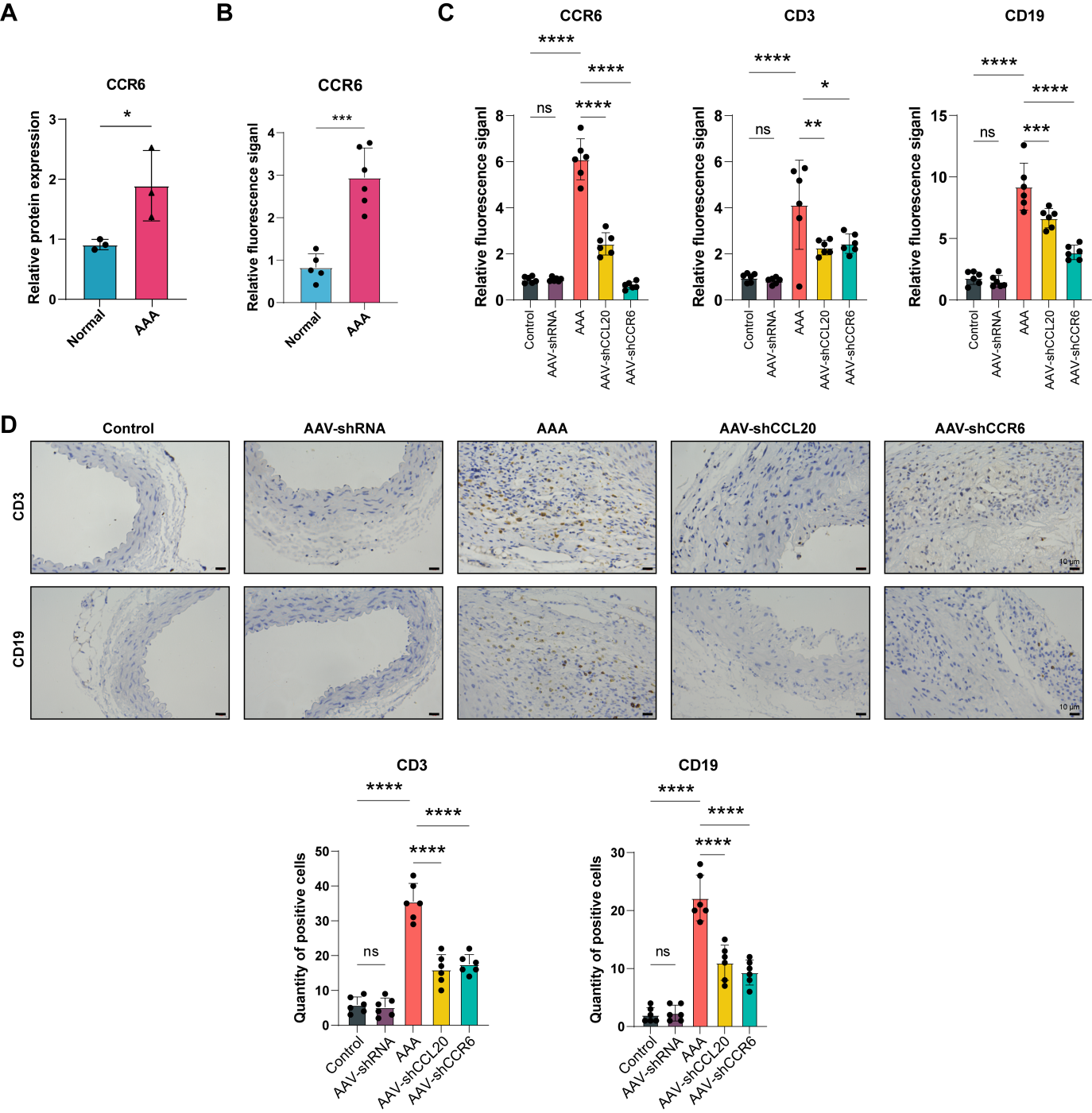


**Supplementary Figure 5.** (A) The relative protein expression level of CCR6 between the Normal and AAA groups. (B) The relative fluorescence signal level of CCR6 between the normal (n=5) and AAA groups(n=6). (C) The relative fluorescence signal level of CCR6, CD3 and CD19 of all groups, n=15 per group. (D) Representative IHC images of CCR6, T cells, and B cells in the aorta tissues of all groups, n=15 per group. Scale bar, 10 μm. **P* < 0.05, ***P* < 0.01, ****P* < 0.001, and *****P* < 0.0001.

**Supplementary Figure.6**


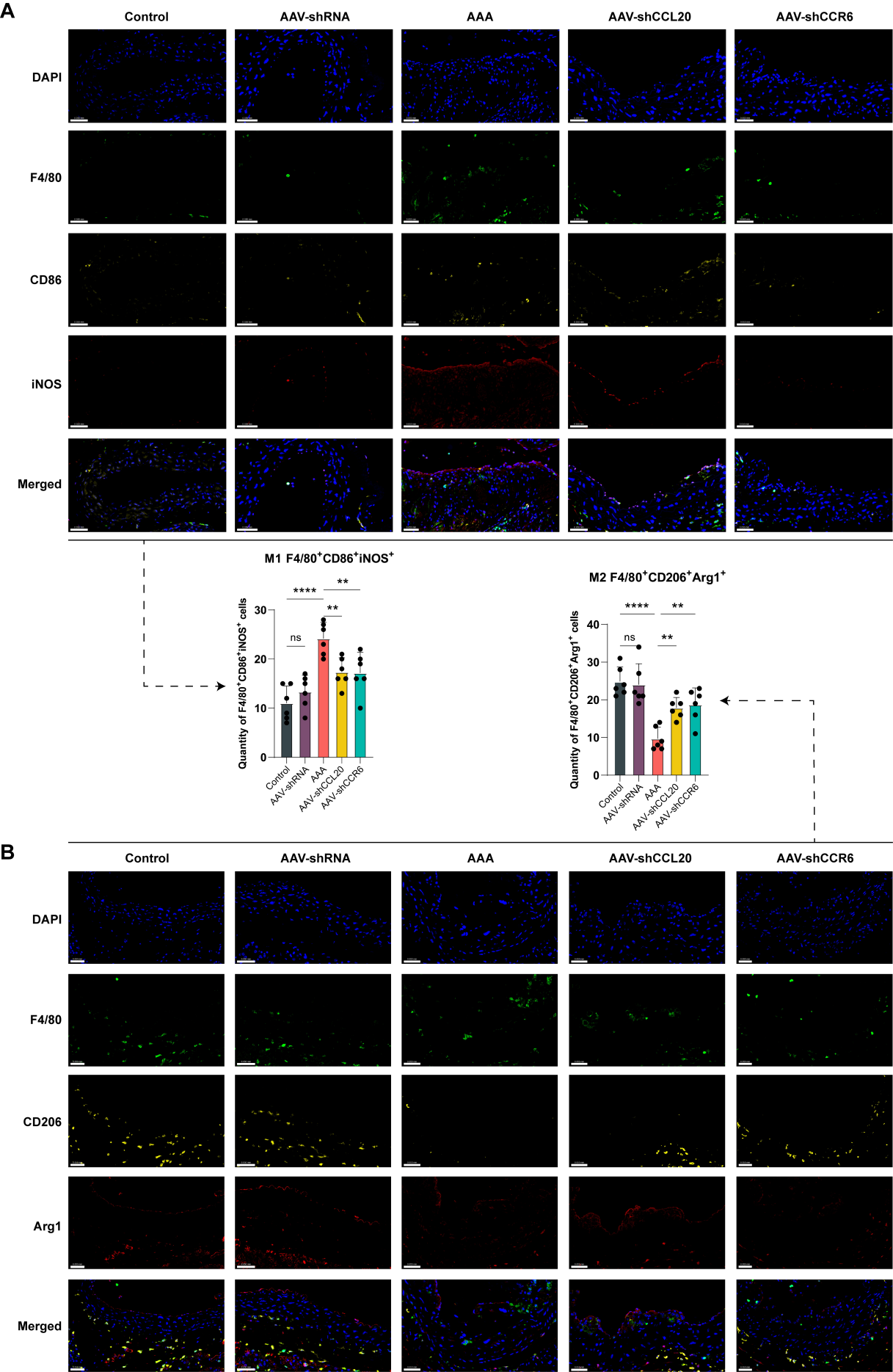


**Supplementary Figure 6.** (A-B) Representative images of IF of M1-like macrophages (F4/80, green; CD86, yellow; iNOS, red) and M2-like macrophages (F4/80, green; CD206, yellow; Arg1, red) of all groups, n=15 per group. ; nuclei were stained with DAPI (blue), scale bar, 50 μm. ** *P* < 0.01, **** *P* < 0.0001.

**1.2 Supplementary Tables**

| **Supplementary Table 1. Demographics and comorbidities of AAA patients and organ donors.** | | | | | | | | | |
| --- | --- | --- | --- | --- | --- | --- | --- | --- | --- |
| Variable | Sex | Age  (y) | Diagnosis/  Comments | Smoking | Drinking | HTN | DM | HLP | CAD |
| AAA1 | M | 66 | AAA | No | No | Yes | No | No | No |
| AAA2 | M | 69 | AAA | Yes | No | Yes | No | No | No |
| AAA3 | M | 58 | AAA | Yes | Yes | No | No | No | No |
| AAA4 | F | 61 | AAA | No | No | Yes | No | No | No |
| AAA5 | F | 71 | AAA | No | No | Yes | No | No | No |
| AAA6 | M | 70 | AAA | Yes | Yes | No | No | No | No |
| Control1 | M | 74 | Organ donors | No | No | Yes | No | No | No |
| Control2 | M | 51 | Organ donors | No | No | Yes | No | No | No |
| Control3 | M | 49 | Organ donors | No | No | No | No | No | No |
| Control4 | M | 56 | Organ donors | No | No | Yes | No | No | No |
| Control5 | M | 60 | Organ donors | No | No | Yes | No | No | No |
|  | Age range | | Mean | Median | | Standard deviation(SD) | | Interquartile range (IQR) | |
| AAA Group | 58-71 | | 65.83 | 67.5 | | 5.27 | | 10 | |
| Control Group | 51-74 | | 58 | 56 | | 9.92 | | 17 | |
| HTN: Hypertension; DM: Diabetes Mellitus; HLP: Hyperlipidemia; CAD: Coronary Artery Disease. | | | | | | | | | |

| **Supplementary Table 2. Demographics and comorbidities of the AAA and Health groups. Related to Figure 4L and M.** | | | |
| --- | --- | --- | --- |
|  | AAA | Health control | *P*-value |
| N | 80 | 79 |  |
| Age (years) | 69.26 ± 7.77 | 60.76 ± 7.38 | 0.066 |
| Women (%) | 6.25% | 19% | 0.086 |
| Aortic diameter (cm) | 5.83 ± 1.09 |  |  |
| Hypertension (%) | 75% | 5.03% | <0.0001 |
| Diabetes Mellitus (%) | 20.98% | 0 | <0.0001 |
| Smoking (%) | 43.75% | 20.25% | 0.0021 |
| Drinking (%) | 37.5% | 13.92% | 0.00097 |
| Hyperlipidemia (%) | 7.5% | 0 | 0.003 |
| CAD (%) | 33.75% | 0 | <0.0001 |
| COPD (%) | 13.75% | 0 | <0.0001 |
| Nominal variables are presented as %. Continuous variables are presented as mean ± SD. P-value refers comparison the AAA and Health group. F: Female; M: Male; HTN: Hypertension; DM: Diabetes Mellitus; HLP: Hyperlipidemia; CAD: Coronary Artery Disease. | | | |

| **Supplementary Table 3. Primer sequences used for real-time qPCR analysis** | | | |
| --- | --- | --- | --- |
| Genes | Species | Sequence (Forward) | Sequence (Reverse) |
| GAPDH | Human | AGGTCGGTGTGAACGGATTTG | GGGGTCGTTGATGGCAACA |
| CCR6 | Human | GAACCCTGTGCTCTACGCTT | CGCATTGTCGTTATCTGCGG |
| IL-8 | Human | ACTGAGAGTGATTGAGAGTGGAC | AACCCTCTGCACCCAGTTTTC |
| IL-1β | Human | CAACAAGTGGTGTTCTCCATGTC | ACACGCAGGACAGGTACAGA |
| TNF-α | Human | GAGGCCAAGCCCTGGTATG | GGGCCGATTATCTCAGC |
| ACC1 | Human | CATTTTCGGTCAGGAAGAATTGC | TGGAAGCATTATTACCACGAAGG |
| FASN | Human | AAGGACCTGTCTAGGTTTGATGC | TGGCTTCATAGGTGACTTCCA |
| PPAR-γ | Human | GCCTCTATCGTCAACAAGGAC | GCAATGAATAGGGCCAGGTC |
| GAPDH | Mouse | AGGTCGGTGTGAACGGATTTG | GGGGTCGTTGATGGCAACA |
| CCR6 | Mouse | TTGTCCTCACCCTACCGTTC | GATGAACCACACTGCCACAC |
| CCL20 | Mouse | AGACAGATGGCCGATGAAGC | TGGATCAGCGCACACAGATT |
